# A new platform for ultra-high density *Staphylococcus aureus* transposon libraries

**DOI:** 10.1186/s12864-015-1361-3

**Published:** 2015-03-29

**Authors:** Marina Santiago, Leigh M Matano, Samir H Moussa, Michael S Gilmore, Suzanne Walker, Timothy C Meredith

**Affiliations:** Department of Microbiology and Immunobiology, Harvard Medical School, Boston, MA 02115 USA; Department of Ophthalmology, Harvard Medical School, Massachusetts Eye and Ear Infirmary, Boston, MA 02114 USA; Department of Biochemistry and Molecular Biology, Pennsylvania State University, University Park, PA 16802 USA

**Keywords:** *Staphylococcus aureus*, Functional genomics, Transposon library, Temperature-sensitivity, Small colony variants, Essential genes

## Abstract

**Background:**

*Staphylococcus aureus* readily develops resistance to antibiotics and achieving effective therapies to overcome resistance requires in-depth understanding of *S. aureus* biology. High throughput, parallel-sequencing methods for analyzing transposon mutant libraries have the potential to revolutionize studies of *S. aureus,* but the genetic tools to take advantage of the power of next generation sequencing have not been fully developed.

**Results:**

Here we report a phage-based transposition system to make ultra-high density transposon libraries for genome-wide analysis of mutant fitness in any Φ11-transducible *S. aureus* strain. The high efficiency of the delivery system has made it possible to multiplex transposon cassettes containing different regulatory elements in order to make libraries in which genes are over- or under-expressed as well as deleted. By incorporating transposon-specific barcodes into the cassettes, we can evaluate how null mutations and changes in gene expression levels affect fitness in a single sequencing data set. Demonstrating the power of the system, we have prepared a library containing more than 690,000 unique insertions. Because one unique feature of the phage-based approach is that temperature-sensitive mutants are retained, we have carried out a genome-wide study of *S. aureus* genes involved in withstanding temperature stress. We find that many genes previously identified as essential are temperature sensitive and also identify a number of genes that, when disrupted, confer a growth advantage at elevated temperatures.

**Conclusions:**

The platform described here reliably provides mutant collections of unparalleled genotypic diversity and will enable a wide range of functional genomic studies in *S. aureus.*

**Electronic supplementary material:**

The online version of this article (doi:10.1186/s12864-015-1361-3) contains supplementary material, which is available to authorized users.

## Background

*Staphylococcus aureus* is a highly adaptable pathogen that is responsible for tens of thousands of serious infections every year in the United States [1-3]. Over 11,000 people die due to antibiotic-resistant *S. aureus* infections (MRSA) [4]. Resistance has emerged in *S. aureus* to all major classes of antibiotics and is expected to be an ongoing problem [1-3]. Many cellular factors in *S. aureus* contribute to antibiotic tolerance and resistance [5,6]. For example, resistance to β-lactam antibiotics is due to the acquisition of an additional penicillin-binding protein (PBP), PBP2a, which has a lower affinity for β-lactams than the other PBPs [7-9]. PBP2a does not act alone, however; many other auxiliary factors are required for phenotypic β-lactam resistance [10-14]. The cell wall stress response and the stringent response are also required for *S. aureus* to mount a robust cellular response to antibiotic-induced stress [15-18]. A better understanding of how these cellular components work together to combat environmental stresses could lead to new strategies for more efficacious dosing of existing antibacterial agents as well as the development of novel therapeutics.

Transposon libraries are powerful tools for probing bacterial physiology [19]*.* In conjunction with next-generation sequencing (NGS) technologies, high quality transposon libraries have been used to characterize essential and conditionally essential genes in a variety of bacterial species [20,21]. Several variations of high throughput transposon insertion site sequencing have been reported, including transposon directed insertion-site sequencing (TraDIS), insertion sequencing (INSeq), high-throughput insertion tracking by deep sequencing (HITS), and transposon sequencing (Tn-Seq) [22-25]. These approaches chiefly vary in the method of transposon delivery, being tailored for a particular species, and in the preparation of library DNA to map transposon insertion sites. Creation of high-density transposon libraries in *S. aureus* has been challenging because its thick cell wall precludes high-efficiency electroporation of DNA containing the transposon, and there are no systems in *S. aureus* for transposon delivery via conjugation [26-28]. Thus, most high coverage transposon libraries in *S. aureus* typically utilize a temperature sensitive plasmid containing the transposon and require high-temperature plasmid-curing steps to remove the plasmid delivery vehicle after transposition has occurred [29-34]. During this curing step, temperature-sensitive transposon mutants may be culled, making it challenging to differentiate essential genes from those that are required for growth at elevated temperatures.

Here we report the design and application of a phage-based transposition method that is compatible with a new NGS protocol and surmounts the many challenges associated with creating high-density transposon libraries in *S. aureus.* In fact, this method is so efficient that we were able to multiplex together transposon constructs with different regulatory elements that have the ability to over- and under-express as well as inactivate any gene in the genome to create a single highly-diverse library with more than 690,000 unique transposon mutants (Table 1). We have used this library to identify genes essential for growth at 30°C and have assessed transposon mutant fitness at a set of low and high temperatures to identify temperature-sensitive and resistant mutants. Nineteen genes identified as essential in previous studies were found to be conditionally-essential, demonstrating growth inhibition at elevated temperatures. In addition, mutants in the menaquinone biosynthesis pathway were found to be significantly enriched at high temperature. Mutations in this pathway have previously been found in small colony variant (SCV) strains isolated from *in vivo* infections that are resistant to antibiotics, and our results suggest that heat stress is one condition that may select for these mutants. The phage-based delivery and insertion site sequencing methodology described here will facilitate comprehensive functional genomic studies of *S. aureus* physiology.

**Table 1:** **Number of reads and transposon insertions from two biological replicates**

**Donor strain**	**Number of reads**	**Unique TA sites hit**
*P*_pen_	3,168,491	115,859
*P*_cap_	2,938,859	105,437
*P*_tup_	4,594,924	130,003
*P*_erm_	4,361,930	111,657
Dual	3,176,044	105,759
Blunt	5,126,052	126,040
Total:	23,366,335	694,755

## Results

### Adapting the transposon system for compatibility with any *Staphylococcus aureus* strain

**Figure 1:**
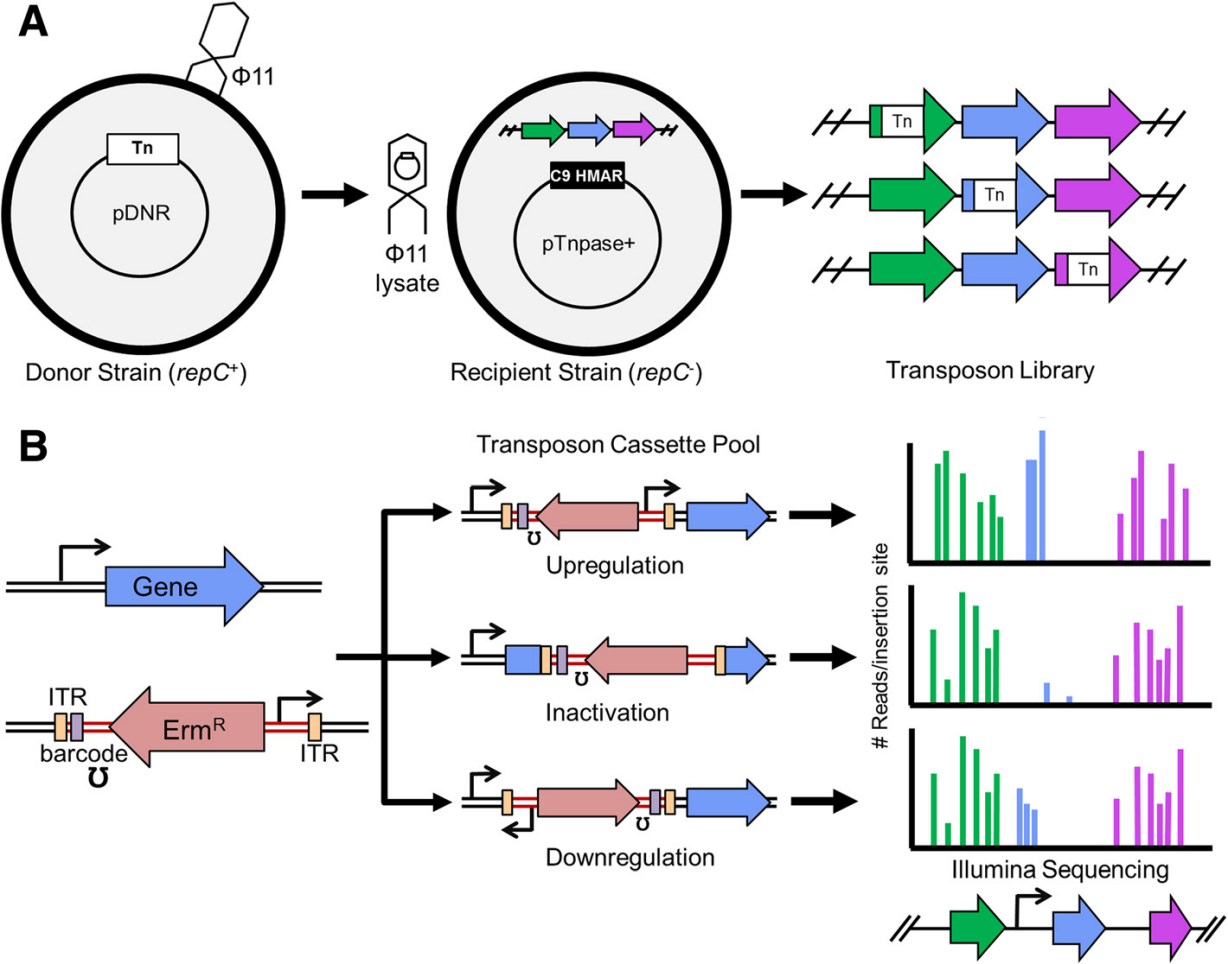
**Strategy for using phage-based transposition to make high quality transposon libraries for NGS sequencing. (A)** The transposon insertion library is made by creating a high-frequency transducing lysate of the transposon cassette that is able to replicate as a plasmid in the donor strain (*repC* ^*+*^). The lysate is mixed with the recipient strain (*repC* ^*−*^) carrying a temperature sensitive plasmid from which the Himar1 transposase is expressed, and erythromycin resistant transposon insertion mutants are selected. **(B)** By fitting the transposon cassette with an outward-facing promoter, genes can be up- or down-regulated, or inactivated if non-essential, in a single library pool. In order to cover a wide range of gene expression levels, different promoter containing transposon constructs can be multiplexed and then identified during NGS sequencing by unique DNA barcodes (purple bar) to collect fitness-gene dose relationships by monitoring read counts.

Recently, we reported a phage-based approach for *S. aureus* that enables high efficiency delivery of transposons [35], but it was not compatible with next generation sequencing. The phage-based transposon system developed previously uses a conditionally replicative transposon donor plasmid, which is moved by generalized phage transduction to a Himar1 transposase-expressing strain that cannot support plasmid replication (Figure 1A). Following transduction, the transposase inserts the transposon into TA dinucleotide sites randomly across the genome. Whereas other transposon systems generate only null genotypes due to gene disruption, the phage-based system was designed to allow over- and under expression of genes as well as inactivation [35]. This was achieved by building a set of transposon donor constructs harboring the mariner inverted terminal repeats (ITR) flanking an erythromycin resistance gene under the control of its own promoter and terminator along with an outward-facing weak, medium or strong promoter. Genes proximal to the insertion site can be upregulated or downregulated to different extents depending on the orientation of insertion and the strength of the promoter (Figure 1B). This phage-based transposition system was used to select for determinants conferring resistance to a panel of antibiotics. It was shown to be useful for nominating antibiotic targets through gene upregulation and for identifying other cellular factors involved in antibiotic resistance through deletion/downregulation [35]. However, in its original format the phage-based system was limited to sequencing single colonies isolated after positive selection on agar plates. Thus, its use as a functional genomics discovery tool was limited and we sought to reengineer it to take full advantage of the power of next generation sequencing.

We first modified the transposase plasmid used in the recipient strain to include *orf5*, the gene encoding the Φ11 *c*I-like repressor. The original phage-based transposon delivery system was used to make mutant libraries in *S. aureus* strains RN4220 and COL [35], and the transposon donor plasmids were designed with a 1 kb Φ11 homology region to stimulate efficient packaging of transposon donor plasmid DNA as concatemers by the phage (Additional file 1: Figure S1A). It proved necessary to integrate the Φ11 *c*I-like repressor (o*rf5*) into the genome to prevent replication and lysis of the recipient strain by the wt Φ11 population that did not package plasmid. Because we wanted to use the phage-based transposition system in other *S. aureus* strains without having to first integrate the *c*I-like repressor into the genome, we moved the *orf5* gene into the transposase plasmid (pORF5) to achieve transposase expression and inhibition of Φ11 replication in a single step (Additional file 1: Figure S1B).

**Figure 2:**
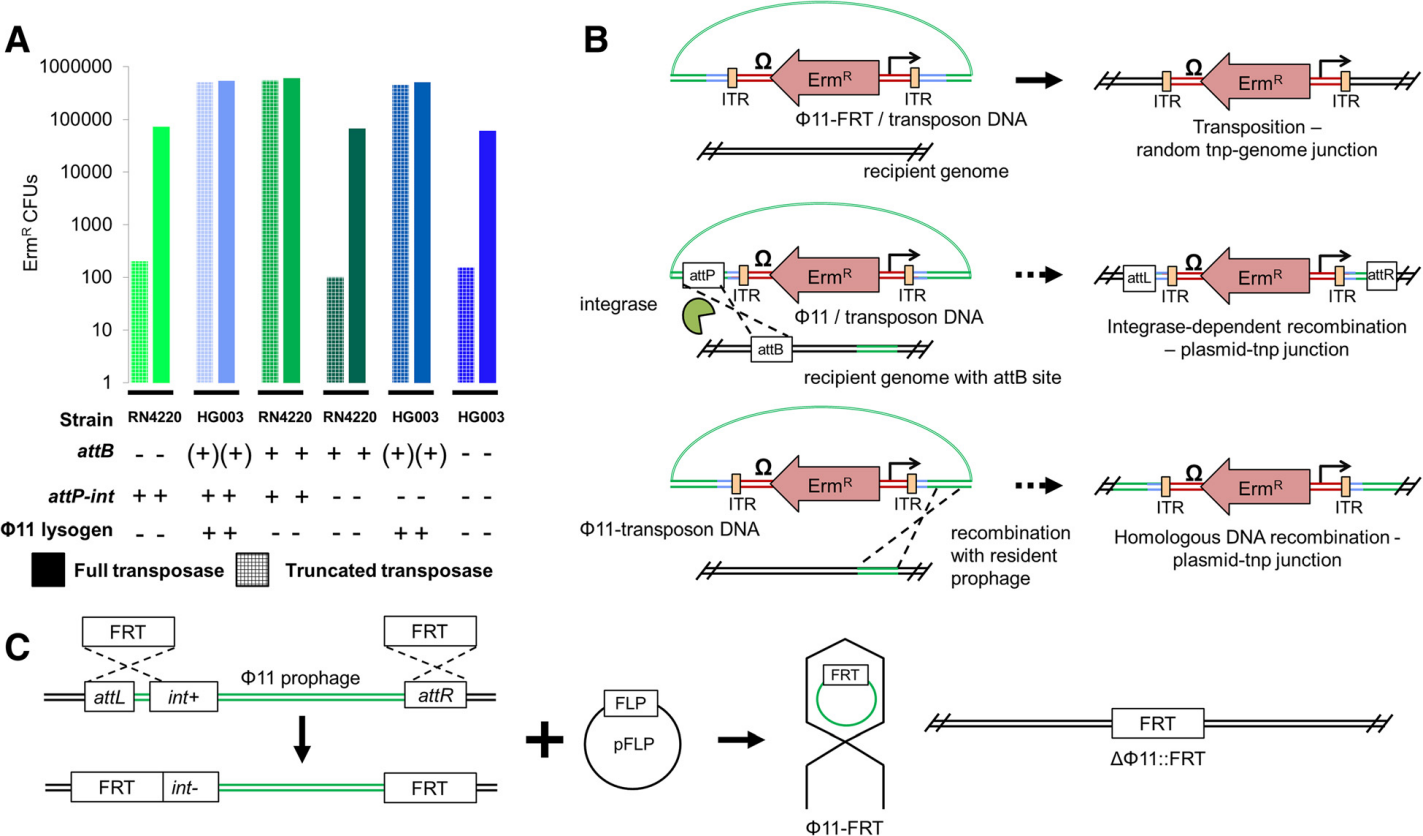
**Elimination of non-transposase catalyzed transposon integration in strain HG003. (A)** The ratio of erythromycin resistant colonies arising from non-transposase catalyzed (hatched) to transposase dependent (solid shading) events was determined in the *S. aureus* RN4220 (green) or HG003 (blue) recipient strain background by comparing the number of colonies resulting from transduction of the transposon into the full transposase or truncated transposase expressing strains (Additional file 1: Figure S1). The presence of the phage attachment site in the bacterial chromosome (*attB*), the phage attachment site in the donor lysate (*attP-int*), and a Φ11 prophage in the recipient is indicated for each combination. **(B)** Putative mechanisms for integration of the erm^R^ cassette of the transposon into the recipient chromosome include transposase catalyzed (top), integrase mediated site specific recombination (middle), and homologous recombination (bottom) of phage-transposon hybrids resolved from concatemeric transposon donor DNA. **(C)** The integrase pathway was blocked by replacing the integrase *(int*) gene and the *attL* sequence with a FRT element by allelic replacement. To cure the resulting prophage, the *attR* site was also replaced with a second FRT site and a phage donor lacking *int-attP* was isolated by introducing the FLP recombinase. In the process, a recipient HG003 strain was generated from which the Φ11 prophage was specifically cured and replaced with a single FRT element, preventing homologous recombination.

We selected HG003 as our strain background for library preparation because a high quality plasmid-delivered library has been made in the same strain and provided a reference for validating our method [29,30]. However, in contrast to RN4220 and COL, a high number of erm^R^ colonies was observed in HG003 even in the absence of functional transposase (<1% for RN4220 versus >90% for HG003; Figure 2A, bars 1 to 4). We initially speculated that phage-transposon hybrid DNA containing an *attP* site was being integrated within a recipient HG003 subpopulation where the chromosomal *attB* site had become available through spontaneous excision of the resident prophage, thus leading to the high background of non-transposase catalyzed events (Figure 2B, middle panel). To confirm this possibility, we introduced the pORF5 transposase plasmid into wild type RN4220, which also contains an available *attB* site. As with HG003, we now observed a high background of non-transposase catalyzed erm^R^ colonies in RN4220 *attB+* (Figure 2A, bars 5 and 6), consistent with a role for phage-mediated *att-*site specific integration in increasing background (Figure 2B, middle panel). To block this pathway, we constructed a strictly virulent transducing phage (Φ11-FRT) by replacing the *attP-int* site with a FRT site from the yeast 2-μm plasmid site-specific recombination system [36,37] (Figure 2C), thereby preventing integration. The use of Φ11-FRT decreased the background of non-transposase-catalyzed transposon integration in RN4220 as fewer erm^R^ colonies were produced by the truncated transposase expressing strain (Figure 2A, bars 7 and 8), but the background in HG003 remained unacceptably high (Figure 2A, bars 9 and 10). We therefore considered a second mechanism for the production non-transposase catalyzed erm^R^ colonies. Homologous recombination between phage-transposon hybrids carrying the erm^R^ cassette and the resident Φ11 prophage in HG003 could also yield erm^R^ colonies (Figure 2B, bottom panel). To determine whether this was occurring, we constructed a strain of HG003 where the Φ11 prophage was specifically removed using the same *att::FRT* exchange strategy employed to create the Φ11-FRT donor phage (Figure 2C). The combination of this recipient strain (HG003 Φ11^−^) with Φ11-FRT packaged transposon donors reduced non-transposase catalyzed erm^R^ background colonies to less than 1% (Figure 2A, bars 11 and 12). With removal of the Φ11 prophage, this strategy now allows us to create high-density transposon mutant libraries with a very low background of non-transposase catalyzed transposon integration using the phage-based transposition system in any strain of *S. aureus* that is transducible by Φ11*.*

### Adapting the phage-based transposon system for Next-Generation Sequencing

**Figure 3:**
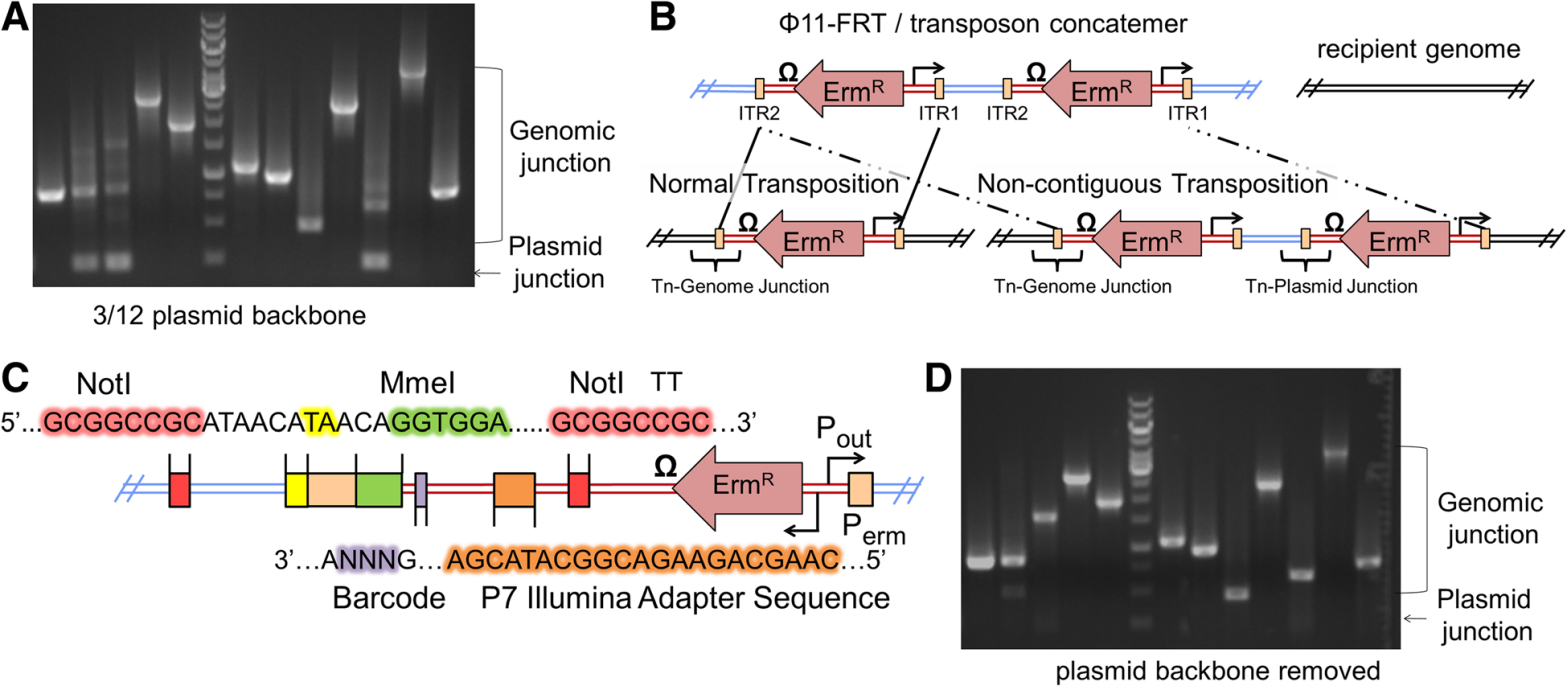
**Reduction of transposon-plasmid junction NGS reads with flanking** ***Not*** **I restriction sites. (A)** Inverse PCR was used to amplify the ITR2 transposon junctions for twelve colonies as has been described [39]. Three out of twelve of these colonies also contained transposon-plasmid junctions (~160 bp DNA band). This ratio increased to seven out of twelve when the canonical ITR sequence was altered to incorporate a MmeI recognition site (Additional file 1: Figure S2). Results are representative of multiple independent experimental replicates. **(B)** The putative mechanism for transposase catalyzed integration of transposon-plasmid junctions may involve engagement of non-contiguous ITR repeats (dashed lines), resulting in chromosomally integrated transposon multimers. In contrast, when both ITR sequences are optimal, contiguous ITRs are most frequently mobilized (solid lines). **(C)** Colors are used to identify the positions of the sequences in this drawing. To selectively remove transposon-plasmid junctions, we introduced two *Not*I sites into the transposon construct that flanked the MmeI modified ITR2. In addition, we included a P7 Illumina sequencing primer site with a unique 3-bp DNA barcode to identify the *P*_out_ promoter that faces outward from ITR1 during NGS sequencing. **(D)** After first digesting gDNA with *Not*I, the transposon-plasmid junction content was substantially reduced in comparison to Figure 3A.

To adapt the transposon system for NGS, modifications to the donor plasmids were required. The initial design included the incorporation of: 1) the P7 Illumina adapter sequence within the transposon cassette to enable Illumina based NGS, 2) unique three base pair barcodes specific to each outward facing promoter element for de-multiplexing after sequencing, and 3) a MmeI site to capture the transposon-genome junction. The MmeI restriction site was embedded within one ITR of the transposon by a single base pair change, and facilitates processing of transposon insertion sites by cutting non-specifically 20-base pairs downstream of its recognition site (16-base pairs downstream of the Himar1 TA dinucleotide insertion site) [22]. Early efforts to prepare and sequence our transposon libraries using the reengineered constructs were plagued by high plasmid-transposon junction read counts, despite the fact that the vast majority of erm^R^ colonies arose via *bona fide* transposition events (Figure 2A). When we used PCR to probe the transposon junctions in isolated transposon mutant colonies, we observed a small population harboring both plasmid- and genomic-transposon junctions (Figure 3A), as previously reported [35]. However, when a single base pair was changed in the canonical ITR DNA sequence to create the MmeI site, the population of transposon insertion mutants containing plasmid-transposon junctions increased to over 50% (Additional file 1: Figure S2). We hypothesized that the MmeI modified base in the ITR was important for recognition by the Himar1 transposase *in vivo*, resulting in a transposase-DNA complex that often failed to engage the initially encountered ITR and instead read through to a downstream non, contiguous ITR within the concatemer (Figure 3B) [38]. The ensuing transposition event would thus capture and introduce the intervening plasmid region into the recipient genome. Therefore, we added two NotI sites to the plasmid, one immediately after the MmeI-modified ITR within the plasmid backbone and the second immediately upstream of the P7 adapter sequence (Figure 3C). Following an added NotI digestion step, the plasmid-transposon junctions could now be selectively removed as described below and in the Additional file 1 (Figure 3D).

**Figure 4:**
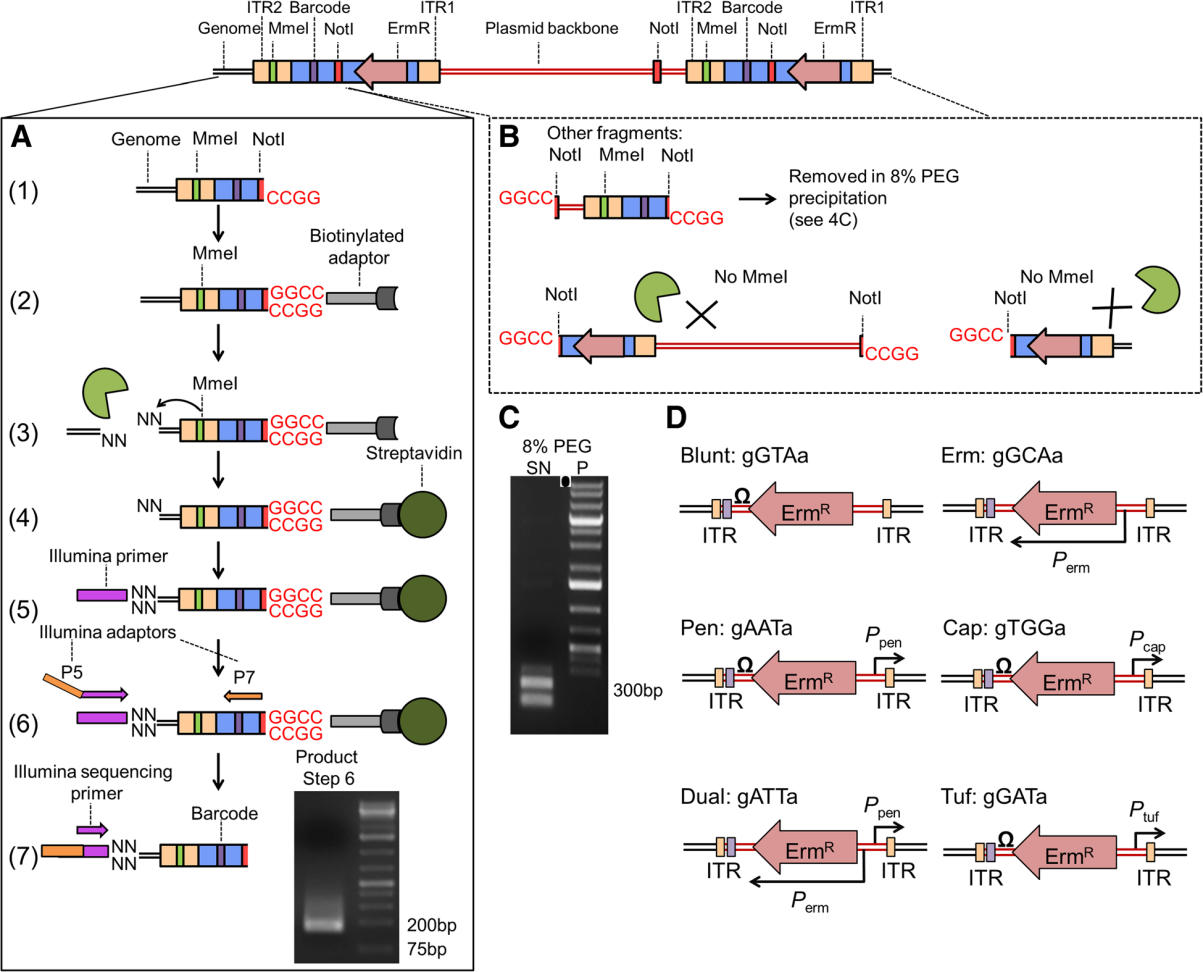
**Protocol for the preparation of a high quality transposon DNA library for NGS. (A)** (1) Genomic DNA is isolated and digested with *Not*I. High molecular weight DNA is selectively precipitated using an 8%PEG + NaCl solution, and transposon-plasmid junctions (106 bp) are removed in the supernatant. A biotinylated dsDNA adapter with *Not*I overhang is ligated (2) before digestion with *Mme*I, which cuts non-specifically 20 bp from its recognition site within ITR2 into the genome to liberate biotinylated-transposon-genome junctions as short DNA fragments (114 bp). (3). Biotinylated fragments are bound to streptavidin beads (4), and an Illumina sequencing primer adapter containing an indexing barcode and *MmeI* compatible ends is ligated (5). Primers annealing to the P7 site and the Illumina sequencing primer adapter sequence (with a P5 site overhang) are used to PCR amplify the transposon-genome junctions (6), agarose gel purified, and submitted for Illumina sequencing (7). NGS reads capture both the 16-bp of flanking genomic DNA as well as the transposon donor specific barcode located between the P7 and ITR2. **(B)** Fragments arising from transposon-plasmid junctions are removed by size selective PEG-NaCl precipitation, while the remaining fragments lack both P7 annealing sites and *Mme*I sites for ligation of the Illumina sequencing primer adapter. These fragments are therefore not amplified in step (6) of 4A. **(C)** By performing the size-selective precipitation on a 1 kb DNA ladder, we show that small 300 bp fragments of DNA are retained in the solution (SN), while larger DNA is precipitated (P). **(D)** Six transposon donor constructs were multiplexed and designed to attenuate expression of genes proximal to the insertion site according to the regulatory elements located at the ends of the transposon backbone. Each donor can be identified from NGS reads by the unique 3 bp barcode.

Our optimized sample preparation procedure for transposon mapping is outlined in Figure 4A, and a detailed version can be found in the Additional file 1. Briefly, genomic DNA is extracted from a pooled transposon library and then digested with NotI followed by a size-selective poly-ethylene glycol (PEG) precipitation to remove liberated plasmid-transposon junctions (Figure 4B) [40,41]. A PCR-based quality control check is performed to confirm that background due to transposon-plasmid junctions is minimal (Additional file 1: Figure S3A). A biotinylated dsDNA adapter containing a NotI compatible overhang is then ligated and the DNA is digested with MmeI. The transposon-genome junctions with 2-base overhangs are bound to streptavidin dynabeads and ligated to an adapter containing the indexing barcode and priming site for the Illumina sequencing primer. Primers containing the P5 and P7 sites are then used to amplify the transposon-genome junctions bound to the streptavidin dynabeads. The fragments are run on a 2% agarose gel to confirm size, gel extracted, and multiplexed with other samples prior to sequencing. Using this protocol, we routinely reduced the amount of contaminating plasmid-transposon reads to less than 1%. To confirm the quality of the DNA insert library for NGS, we developed a quality control procedure for determining level of background due to transposon-plasmid junctions (Additional file 1: Figure S3B). This strategy, which utilizes restriction enzyme digestion followed by size-selective precipitations, can be generalized to other bacterial species and transposon library sequencing strategies to prepare transposon libraries for NGS.

### Creation and sequencing of a transposon library

Using the strains and methods described above, we created a 2 million colony transposon library using six different transposon donor constructs containing promoters of different strengths. The donor constructs contained transposon cassettes with a panel of different outward facing promoters (in order of increasing strength *P*_*erm*_*, P*_pen,_*P*_cap,_ and *P*_tuf,_), a construct with the *P*_pen_ outward-facing promoter on one end and the erythromycin-resistance gene promoter (*P*_erm_) on the other but no intervening transcriptional terminator (Dual), and a transposon construct with no outward facing promoter elements (Blunt) (Figure 4D). Library cultures were grown for 12-13 generations in tryptic soy broth (TSB), harvested between an OD of 1 and 1.5, and genomic DNA isolated. After processing samples for NGS (Figure 4), Galaxy was used to separate the data by sample index and donor construct barcodes (Figure 4D) [42-44]. The data were filtered by Illumina quality score for high quality reads and mapped with Bowtie (ver1.1) to the *S. aureus* NCTC8325 reference genome [45]. Full details of the bioinformatics analysis are available in the methods section.

We used two biological replicates for our analyses and computationally de-multiplexed the data by transposon construct based on their unique barcode (Figure 4D). For each of the donor constructs, we obtained 3 to 5 million reads that could be mapped to TA insertion sites. Depending on the constructs, insertions were identified in 105,000 to 130,000 of the ~270,000 unique TA sites in the *S. aureus* genome (Table 1). On average, 3,897,389 ± 834,906 reads hit 115,792 ± 9,416 TA sites for each of the six transposon constructs, with 36,794 TA sites in common between all six of the donor constructs, and 208,372 TA sites covered by at least one donor construct.

### Identification of essential genes

To validate the quality of the library and assess whether insertions due to individual transposon constructs could be reliably analyzed, we used the data obtained to identify essential genes. We first compared the number of reads per gene for each transposon donor construct using principal component analysis and found that the transposon constructs with outward-facing promoters co-clustered away from the promoterless (Blunt) and weakest promoter (*P*_*erm*_) constructs (Additional file 1: Figure S4A). This indicates that transposon insertions in to the same TA site by different transposon constructs are differentially tolerated due to polar effects on downstream genes. This results in different numbers of reads mapping to each gene depending on the identity of the transposon construct. However, because transposon constructs containing an outward-facing promoter (Dual, *P*_*pen*_, *P*_*cap*_, and *P*_*tuf*_) clustered together, the number of reads mapping to each gene for each of these constructs will be more similar to each other than to Blunt or *P*_*erm*_. Because of the similarity between the constructs with an outward-facing promoter, the data from these constructs was combined for the gene essentiality analysis.

We then identified essential genes using a recently published method, EL-ARTIST [46,47]. This method uses a hidden Markov model (HMM) to categorize genes as essential, non-essential, or containing essential domains (domain essential). Because this method uses local context of the TA site as well as the number of reads in that TA site to determine essentiality, it lessens the impact of the intrinsic variability in the data and allows us to robustly ascertain the importance of every gene to cellular survival [46,47]. There were 261 genes found to be essential in all three categories (Blunt, *P*_*erm*_*,* and other promoter constructs), the Blunt construct had the greatest number of essential genes (321 genes), while the *P*_*erm*_ construct had slightly fewer (295 genes), and the other promoter constructs had the least (277 genes) (Additional file 1: Figure S4B). We were curious about the differences between the Blunt construct and constructs containing promoter elements. When a transposon inserts into the coding sequence of a gene, it not only interrupts the expression of that gene, but can also disrupt the expression of other genes due to polar effects. It is possible that some of these polar effects are abrogated by the promoters in the *P*_*erm*_ and other promoter constructs, which allow downstream genes to be expressed from the promoter in the transposon instead of the native promoter. If this is true, then the genes found to be essential with the Blunt construct, but non-essential with the other constructs, should be next to or near other essential genes. Of 20 essential genes found using the Blunt construct that were non-essential for the *P*_*erm*_ construct and the other promoter constructs, 13 were immediately proximal to another essential gene. Another five were immediately proximal to a domain essential gene, and three of these were followed by another essential gene. Only two genes were not adjacent to an essential or domain essential gene. These data suggest that when a transposon containing an outward-facing promoter inserts into a region containing many essential and domain essential genes, the insertion may result in a less lethal phenotype than the standard transposon construct. Due to these differences and to compare our data with previous studies which did not have constructs containing promoters, we chose to use only the Blunt construct for subsequent analyses.

**Figure 5:**
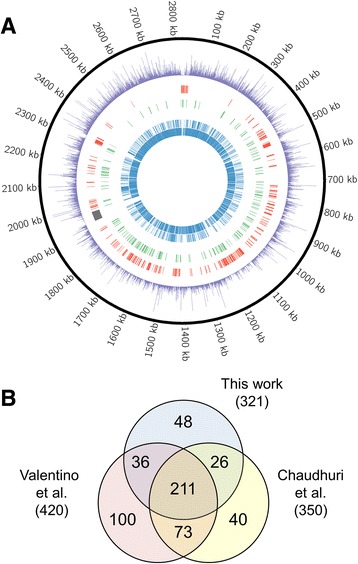
**The essential gene sets at 30°C. (A)** The Circos program was used to map transposon insertion sites across the genome, with a histogram depicting the number of reads per TA site in purple. The innermost blue rings depict the locations of non-essential genes for which a fitness cost was not observed, while the outermost red ring depicts those genes identified as essential. The middle green ring represents those genes that were found to be domain essential. **(B)** Venn diagram comparing our essential gene list to Chaudhuri *et al*. and Valentino *et al* [29,33]. There is extensive overlap between the three studies. 211 genes were classified as essential in all three works. These represent a core set of genes required for cell growth regardless of experimental and analytical variations.

The Blunt construct, which is not fitted with gene expression modulating elements, does not include promoters that affect proximal gene expression and is most similar to previously investigated transposons [29-34]. Therefore, we used it for the rest of the analyses so that we could compare our results to previous studies. In two biological replicates, there were ~126,000 unique insertion sites due to the Blunt construct. Using the Circos program to display the number of reads per TA site [48], we visually confirmed randomly distributed insertions throughout the length of the genome (Figure 5A). Of the 2723 coding regions of the genome that are not part of the Φ11 family lysogen, EL-ARTIST called 2212 non-essential, 190 domain essential, and 321 essential (Additional file 2).

We compared these results to two previous studies that identified essential genes in *S. aureus* based on transposon mutant analysis, one in the HG003 genetic background [29] and the other in SH1000 [33]. Both strains are derived from the NCTC 8325 parent strain. The transposon libraries were made, grown, and analyzed using different methods, but there were 247 essential genes in common between this study and the previous study in HG003, and 211 genes in common between all three studies (Figure 5B). There were 73 genes identified as essential in both previous studies that did not meet our cutoffs for essentiality. Of these, two are no longer considered to be genes, eight were in our list of non-essential genes, and 63 were found to be domain essential. Because the previous studies were done using transposon libraries made using a high-temperature plasmid-curing step, we checked to see if any of these genes would be identified as essential by EL-ARTIST at 43°C. Of the 63 domain essential genes, nine were identified as essential at high temperatures, with nine of the remainder found to be domain essential as at 30°C. One gene, SAOUHSC_01028, encoding the phosphor-carrier HPr was found to be non-essential at 43°C. Transposon insertions in domain essential genes are likely to result in cells that are weak and/or have growth defects, and depending on the stringency of the method of analysis, these mutants may be mistakenly classified as essential. In addition, at the more stressful high temperatures, some of these weakened mutants may no longer be able to survive. Finally, of the eight genes we found to be non-essential that were previously identified as essential, four became essential at high temperatures, and one became domain essential. The four genes that became essential at 43°C include genes known to be important for high temperatures growth such as the heat shock genes, *dnaJ* (SAOUHSC_01682) and *dnaK* (SAOUHSC_01683), as well as *sepF* (SAOUHSC_01154) which is discussed below. However, overall there was substantial agreement between our essential gene list and the lists generated in previous studies despite analytical and experimental differences (different growth media, growth temperature, and/or strain background) (Additional file 2). The 211 genes found to be essential in all studies comprise a core set of essential genes required for cell survival regardless of the experimental and analytical variations used to identify them.

### Identification of genes important for growth at different temperatures

Because the phage-based method for transposon delivery does not involve a high temperature plasmid curing step, mutant libraries retain insertions in temperature sensitive genes. Hence, after validating the transposon library grown at 30°C, we identified transposon insertions that confer temperature-sensitive and resistant phenotypes by comparing mutant fitness at 16°C, 23°C, 37°C, and 43°C to that at the reference temperature 30°C. At each temperature the library was grown for 12-13 generations and the number of reads due to insertion of the Blunt transposon in each gene was compared to the number of reads obtained after outgrowth at 30°C. Two biological replicates were carried out for each condition and the data were analyzed using the Mann-Whitney U test, correcting for multiple hypothesis testing with the Benjamini-Hochberg procedure [30]. Genes showing a greater than five-fold change (increase or decrease) in the number of reads at the test temperature compared to the 30°C control were considered significant if the corrected p-value was less than 0.05. A full list of affected genes can be found in Additional file 3.

At 16°C and 23°C, only one gene, SAOUHSC_01857, contained a significantly different number of reads due to transposon insertions compared to the 30°C control, with the number of reads being enriched six-fold and five-fold, respectively. At 37°C, reads due to transposon insertions were found to be enriched in five genes involved in branched chain amino acid degradation, *ilvE* (SAOUHSC_00536), SAOUHSC_01611, *bkdA2* (SAOUHSC_01612), *bkdA1* (SAOUHSC_01613), and SAOUHSC_01614 (SAOUHSC_01611, *bkdA1, bkdA2,* and *ilvE* meet significance cutoffs) (Additional file 1: Figure S5). Branched chain fatty acids are built from branched chain acyl-CoA primers, which in turn are derived from the degradation of branched chain amino acids [49]. Branched chain fatty acids increase membrane fluidity upon incorporation into phospholipid bilayers [50]. In *Bacillus subtilis,* enzymes that catalyze the degradation of branched-chain amino acids are induced during cold shock and are speculated to increase membrane fluidity by promoting the incorporation of branched chain fatty acids into membrane lipids [51]. In our data there is a bias against transposon insertions in the branched chain amino acid degradation pathway at temperatures lower than 37°C, in agreement with studies in *S. aureus* and *Listeria monocytogenes* implicating branched chain fatty acids in growth at low temperatures [50,52]. Two genes, SAOUHSC_01154*,* encoding SepF, and the hypothetical protein likely co-transcribed with it, SAOUHSC_01153, proved to have significantly fewer reads at 37°C. SepF is a protein of unknown function thought to be involved in cell division, and was previously identified as essential in *S. aureus* [29,31-34,53], but these studies show it is essential for survival only at temperatures greater than 30°C.

**Figure 6:**
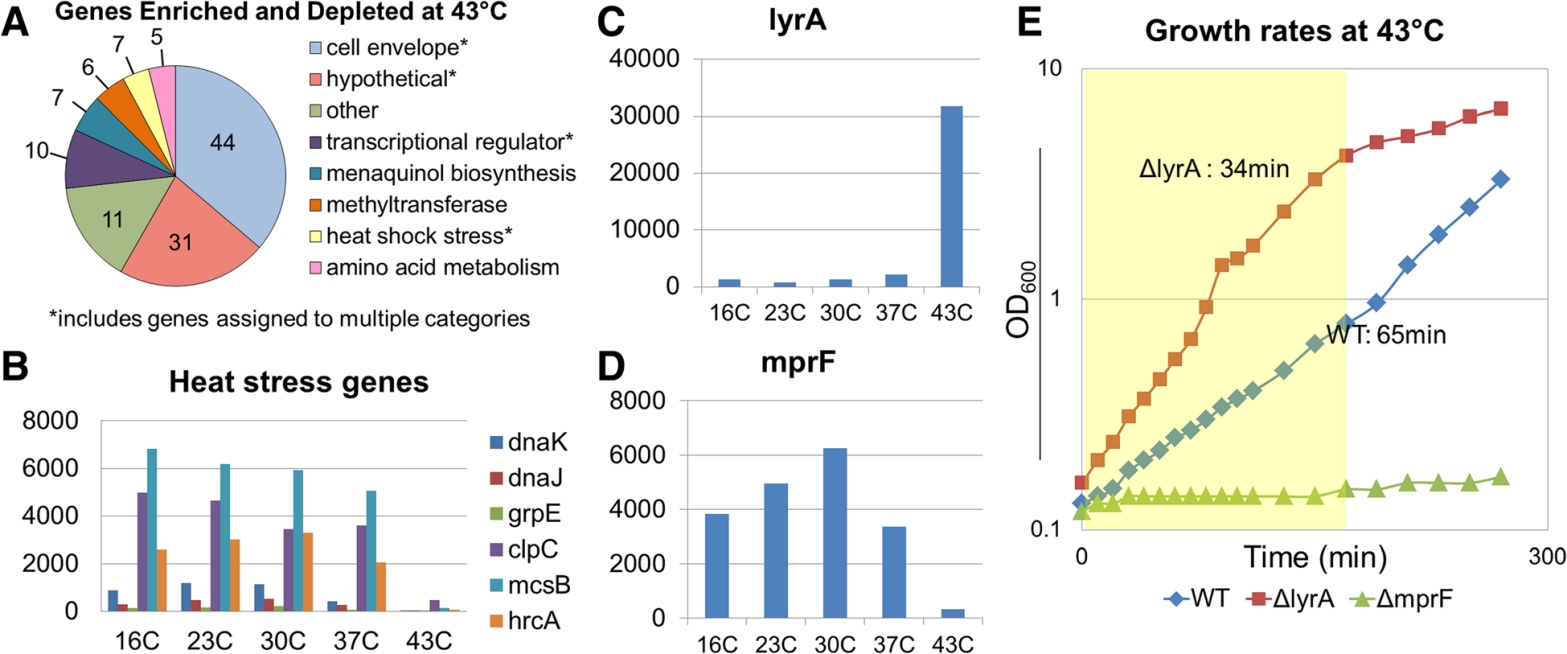
**Genes that influence fitness at high temperature. (A)** Genes contributing to fitness at 43°C were classified by cellular function. Genes associated with the cell envelope constituted the largest fraction of the hits. **(B)** Six genes known to be associated with the heat-shock response were found to be temperature-sensitive. The number of reads in each gene normalized to 5 million sample reads is shown at the various temperatures tested. Loss of *lyrA* and *mprF* was found to have opposite phenotypes at 43°C, with an increase **(C)** and decrease **(D)** in fitness being observed, respectively. **(E)** To validate these phenotypes, null mutants in *S. aureus* HG003 were grown to mid-log phase, diluted, and grown at 43°C. While Δ*mprF* did not grow, confirming temperature sensitivity, the Δ*lyrA* strain grew at nearly twice the rate of WT.

A significant number of genes were found to be affected by transposon insertion when grown at 43°C, with 42 being enriched and 77 being depleted. Because this method for creating transposon libraries does not involve a high-temperature plasmid curing step, we expected to retain many temperature-sensitive mutants. We used two methods for identifying temperature-sensitive mutants represented in the library. The first was the Mann-Whitney U analysis used for every temperature. For the second analysis, we used the 43°C data and the essential gene analysis described in the previous section to identify genes essential at 43°C that were not identified as essential at 30°C. We confirmed that the number of reads mapping to these genes had decreased at 43°C from 30°C. We compared these genes to other essential genes lists [29,31-34], and identified 19 genes that had been annotated as essential in at least two other transposon library analyses, but were only temperature-sensitive with our method (Table 2). To further analyze the temperature sensitive gene subset, we classified them according to cellular function (Figure 6A). Because protection from temperature stress relies on the coordinated response of many different pathways regulated by signaling systems and alternative transcription factors, we were not surprised to find that 11 regulatory genes were significantly depleted at 43°C, including *yycH* (SAOUHSC_00022) *and yycI* (SAOUHSC_00023), which were identified as essential in another transposon library [29]. The *yycH and yycI* genes negatively regulate the two component system, *walKR,* which is a major regulator of peptidoglycan metabolism that controls autolytic activity [54,55]. Deletions of *yycH* and *yycI* result in upregulation of w*alKR*, which induces cell wall defects [54,56]. As *walKR* positively regulates autolytic activity [57], increased autolysis of peptidoglycan could explain how derepression of *walKR* produces a temperature-sensitive phenotype. We also found, as expected, that many genes implicated in the heat shock response were required for survival at 43°C. These included the chaperones *dnaK* (SAOUHSC_01683) and *dnaJ* (SAOUHSC_01682) [58], the transcriptional regulator *hrcA* (SAOUHSC_01685) [59], the protease *clpC* (SAOUHSC_00505) [60], and *mcsB* (SAOUHSC_00504; also known as *yakI*), an arginine phosphotransferase that negatively regulates the stress response repressor *ctsR* [61,62]. GrpE (SAOUHSC_01684), which acts in a complex with DnaK and DnaJ, narrowly missed our cutoffs (Figure 6B) [63].

**Table 2:** **Genes with a growth defect at 43°C that were previously identified as essential**

**Category**	**Gene locus**	**Name**
Cell envelope	SAOUHSC_01154	*sepF* [29,31-34]
	SAOUHSC_01359	*mprF* ^*^ [31,33,34]
	SAOUHSC_01627	putative lipopprotein [29,31-34]
	SAOUHSC_01739	*lytH* [33,34]
	SAOUHSC_01759	*mreC* [31,34]
	SAOUHSC_02319	*rodA* [31,34]
	SAOUHSC_01827	*ezrA* [29,31-34]
	SAOUHSC_02337	*murA* [33,34]
	SAOUHSC_02571	*ssaA* [29,31-33]
	SAOUHSC_01361	*lcpA* ^*^ [31,34]
Protein folding	SAOUHSC_01682	*dnaJ* [29,31-34]
	SAOUHSC_01683	*dnaK* [29,31-34]
	SAOUHSC_01684	*grpE* [29,31-34]
Other	SAOUHSC_00803	ribonuclease R[31,34]
	SAOUHSC_00015	DHH subfamily protein [29,31,33,34]
	SAOUHSC_00892	general stress protein 13 [31,32,34]
	SAOUHSC_01751	*ruvA* [31-34]
Unknown	SAOUHSC_00760	hypothetical protein [33,34]
	SAOUHSC_00788	hypothetical protein [31,33,34]

*mprF aiso annotated as fmtC, and lcpA also annotated as msrR.

The largest category of genes with significant changes in number of reads when grown at 43°C included those involved in cell envelope related processes (46 genes). This category includes genes involved in peptidoglycan (PG) biosynthesis, cell shape/division, membrane lipid composition, transcriptional regulation of cell-envelope genes, and predicted membrane proteins (Figure 6A). Transposon insertions into biosynthetic genes directly involved in PG biosynthesis were notably depleted, including *pbp3* (SAOUHSC_01652) and *pbp4* (SAOUHSC_00646), which encode cell wall transpeptidases [64-66], one of two alleles of *murA* (SAOUHSC_02337) involved in PG precursor biosynthesis [67], *alr1* (SAOUHSC_02305) which encodes one of two alleles of alanine racemase [68], and SAOUHSC_01739, which encodes an *amiC*-like peptidoglycan amidase [69]. Insertions in several genes involved in cell shape/cell division were also affected at 43°C. For example, reads due to transposon insertion were depleted for *rodA* (SAOUHSC_02319), *mreC* (SAOUHSC_01759), and *mreD* (SAOUHSC_01758)*,* which encode scaffolding proteins that coordinate PG biosynthesis and influence cell shape [70-73], and also for *ezrA* (SAOUHSC_01827), which is thought to coordinate Z-ring functions with PG synthesis [74], *ftsH* (SAOUHSC_00486) [75], *sepF* (SAOUHSC_01154) [53], *gpsB* (SAOUHSC_01462) [76]*,* and SAOUHSC_01857. SAOUHSC_01857 encodes a 1200 amino acid FtsK-like protein suggested to be involved in chromosome localization [77]. As noted above, insertions in this gene conferred a growth advantage at 16°C and 23°C, but are deleterious at high temperature. This gene was identified in other studies as essential in *S. aureus* [29,31-34]. The putative cell wall teichoic acid ligases, *lcpA* (SAOUHSC_01361) and *lcpB* (SAOUHSC_00997), were also depleted at 43°C, consistent with the vital role of wall teichoic acids in orchestrating PG assembly and maintaining envelope integrity [13,78,79]. The integral membrane protein MprF (SAOUHSC_01359), which attaches lysine to phosphatidylglycerol, was also important for fitness at high temperatures [80].

**Figure 7:**
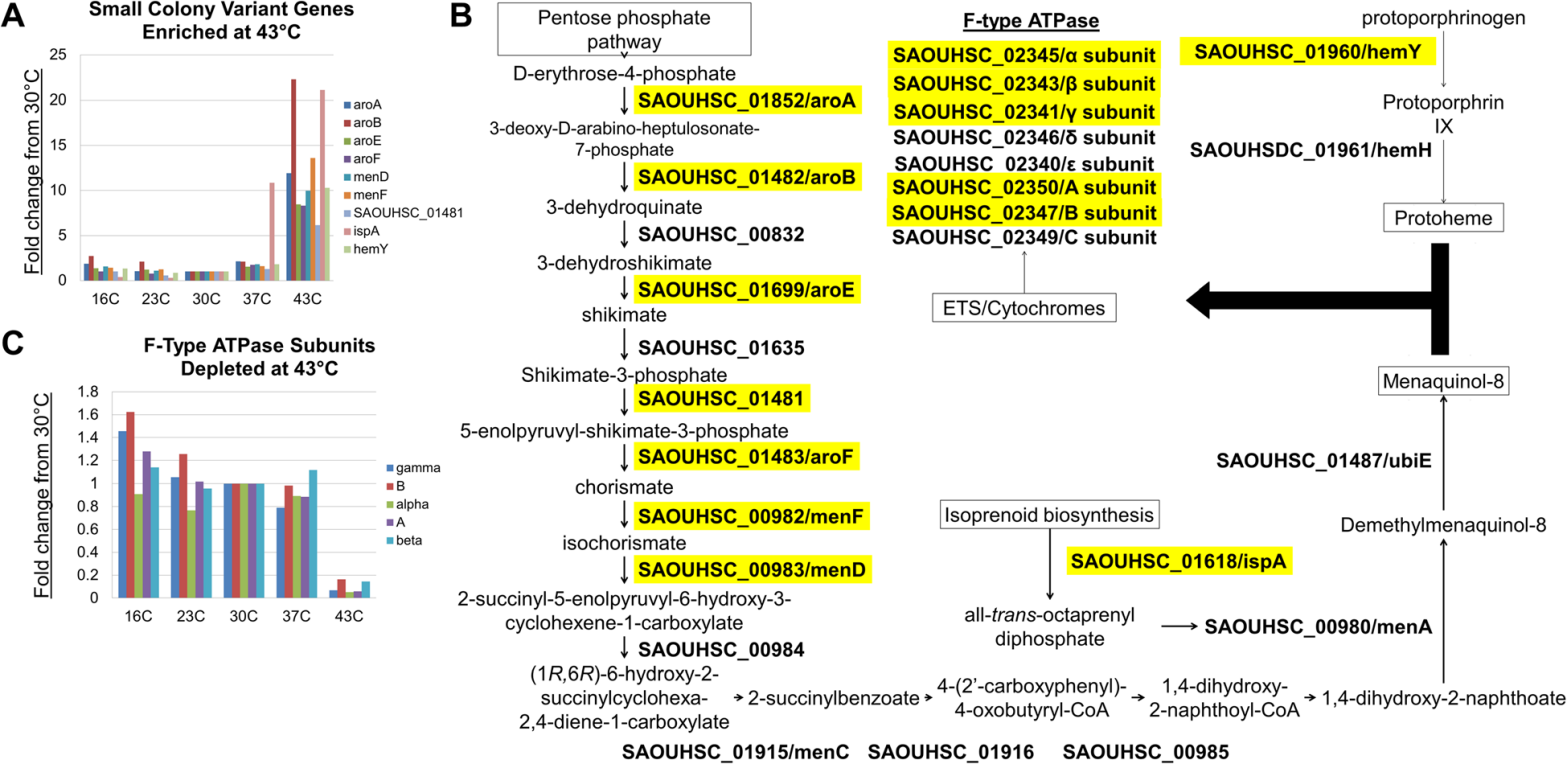
**The electron transport system influences sensitivity to high temperatures. (A)** Nine genes were identified as growth advantaged at 43°C in comparison to 30°C. The number of reads per gene normalized to 5 million reads is shown for each temperature. **(B)** Pathway analysis revealed that this subset of genes (highlighted in yellow) is involved in the biosynthesis of components within the electron transport system, including protoheme and menaquinones. **(C)** Blocking the electron transport system at the F-type ATPase level, however, decreased fitness at 43°C (genes highlighted in yellow). The number of reads per gene normalized to 5 million reads is shown for each temperature.

In addition to the mutants that were depleted at 43°C, we identified a number of processes for which disruption resulted in a significant growth advantage compared to growth at 30°C (Figure 7A). Pathway enrichment analysis using BioCyc [81] showed that the number of reads due to transposon insertion were significantly enriched in seven genes in the aromatic amino acid and menaquinol biosynthetic pathways (SAOUHSC_01481, *aroB:* SAOUHSC_01482, *aroF:* SAOUHSC_01483, *menF:* SAOUHSC_00982, *menD:* SAOUHSC_00983, *aroE:* SAOUHSC_01699, and *aroA:* SAOUHSC_01852) (Figure 7A). In addition to producing phenylalanine and tyrosine, the aromatic amino acid pathway provides precursors (chorismate) for menaquinone biosynthesis (Figure 7B) [82]. Menaquinones are isoprenylated electron transport chain cofactors embedded in the membrane that are necessary for oxidative phosphorylation [83]. Insertions in *ispA* (SAOUHSC_01618), which encodes a putative geranyltransferase, were also enriched at 43°C. Geranyltransferases initiate the condensation of isoprenoid units into longer chains used in the synthesis of menaquinones, and loss of *ispA* likely decreases the pool of menaquinones (Figure 7B). We also identified insertions in *hemY* (SAOUHSC_01460), a gene involved in the production of protoheme [84]. Loss of these factors is thought to shift *S. aureus* growth towards an anaerobic metabolic regime, which markedly impacts *S. aureus* membrane physiology and generates small colony variants (SCV) [85,86]. The observed growth advantage could also be related to more efficient anaerobic catabolism at high temperature, and/or decreased oxidative stress. Five subunit genes in the F-type ATPase involved in electron transport were found to be essential at 43°C (Figure 7C): α-subunit (SAOUHSC_02345), β-subunit (SAOUHSC_02343), γ-subunit (SAOUHSC_02341), A subunit (SAOUHSC_02350), and B subunit (SAOUHSC_02347) (all were significantly depleted except the β-subunit), suggesting the electron transport system (ETS) mutations can have opposing effects on fitness at elevated temperatures depending on which step in the ETS is blocked. Among cell envelope-related genes, reads due to transposon insertion were notably enriched in *lytD* (SAOUHSC_01895), encoding a putative β-N-acetylglucosaminidase [87], and *lyrA* (SAOUHSC_02611), a polytopic membrane protein. Disruptions in *lyrA* were previously shown to increase lysostaphin resistance and are lethal when wall teichoic acid biosynthesis is blocked [30,88], and both these phenotypes are consistent with an important role for lyrA in cell envelope biogenesis.

To confirm the results of our automated transposon insertion analysis pipeline, we constructed and measured the growth rates of null mutants of *lyrA* (significantly overrepresented at 43°C) and *mprF* (significantly depleted at 43°C) (Figure 6C and 6D). While there were no differences in growth of the mutants compared to wild type at 30°C, growth related phenotypes became apparent at 43°C after 5 to 6 generations of outgrowth (Additional file 1: Figure S6). The wild type strain grew more slowly at 43°C than at 30°C (doubling times were 65 min and 37 min, respectively), the Δ*mprF* strain ceased dividing altogether, and the doubling time of the Δ*lyrA* mutant decreased to 34 minutes at 43°C from 41 minutes at 30°C (Figure 6E and Additional file 1: Figure S6). These results confirm the changes in fitness that were deduced from the changes in read number due to transposon insertion in *lyrA* and *mprF* at 43°C.

## Discussion

### Adaptation of the transposon library for NGS

We have adapted a phage-based transposition method to reliably create large and diverse transposon libraries that can be analyzed using high- throughput Illumina sequencing. The high background due to non-transposase-catalyzed recombination in the HG003 strain was reduced to negligible levels by removing the Φ11 family prophage and using a strictly virulent transposon donor phage (Φ11-FRT) for transduction. Other strains that harbor Φ11 family prophages, such as *S. aureus* Newman [89], may have a similar background problem that makes removing the Φ11 family prophage necessary for working in these genetic backgrounds. Fortunately, many commonly used *S. aureus* strains, including RN4220, COL, USA300, and MW2, do not harbor highly similar prophages and need not be reengineered. Indeed, we have successfully constructed ultra-high density transposon libraries in several other *S aureus* strains, including the USA300 and MW2 backgrounds, using the same tools described here (M. Santiago, T. Meredith, and S. Walker, unpublished data).

To realize the full potential of the phage-based delivery system for transposon library generation, we optimized both the donor constructs and the NGS sample preparation procedure. The donor constructs were modified to include two NotI sites so that a straightforward digestion step could be used to reduce plasmid-transposon junction background (Figure 4). While plasmid-transposon junctions are particularly problematic in our case due to the generation of concatemeric DNA by the phage, many *in vivo* transposon delivery vehicles suffer from some degree of transposon donor plasmid retention. Plasmid-transposon junctions consume valuable NGS read counts and the lack of sequence diversity can affect quality scores for the entire sample. We demonstrate that plasmid contamination can be solved by introducing dual restriction enzyme sites flanking the plasmid-transposon junction. Preprocessing genomic DNA samples with NotI allowed us to replace shearing methods with MmeI digestion, a strategy that simplifies library preparation [90]. Moreover, because transposition efficiency is high and the NGS library preparation protocol is robust, we are able to multiplex several different transposon constructs in the same library. By adding a DNA barcode that specifies the gene expression regulatory element in the transposon construct, the roles of over- and under- expression of genes as well as inactivation can be assessed under a given condition during data analysis. For functional genomics and systems level analyses, the ability to capture over- and under-expression genotypes is particularly important because for the first time we will be able to probe the relationship of essential genes to a given cellular process through over/under expression of essential genes.

### Gene essentiality at a set of temperatures

The power of the redesigned phage-based transposition system was demonstrated through the preparation of a transposon mutant library containing ~694,000 independent insertions in the *S. aureus* HG003 strain background (Additional file 2). We identified essential genes at 30°C, and found good agreement between our essential gene list and two other lists published previously, although 63 genes previously annotated as essential were classified by us as domain essential [29,33]. Taking advantage of the fact that the phage-based transposon delivery method does not involve a high temperature plasmid curing step, we also carried out a study of the genetic factors involved in withstanding temperature stress. Many genes responsive to heat- or cold-shock have been identified in *S. aureus* based on transcriptome data [91], but a systematic and comprehensive analysis of the fitness of temperature-sensitive or resistant mutants has not been reported. We assessed the importance of each gene at five different temperatures based on enrichment or depletion of corresponding NGS reads. While reads per gene were largely unchanged compared to the 30°C control between 16°C, 23°C, and 37°C, reads for 119 genes were significantly affected at 43°C (Additional file 3).

In addition to genes previously linked to heat shock and stress responses, we also found that many genes involved in cell envelope processes contribute to fitness at elevated temperature. Some genes in which reads were depleted at 43°C, such as *pbp3* and *pbp4*, directly participate in crosslinking PG [64,65], but others play less understood roles in PG synthesis. For example, our studies have implicated *rodA*, *mreC*, and *mreD* in withstanding temperature stress, as reads in all three genes were depleted by ~50-fold at 43°C (Additional file 3). In rod-shaped organisms, different biosynthetic machines are dedicated to septal and side wall PG synthesis. RodA, MreC, and MreD are components of the machine that makes side wall PG, while FtsZ, FtsL, and Div1B interact with FtsW as part of the PG biosynthetic machinery at the septum [70,92-94]. *S. aureus* is a coccoid organism and is not known to have distinct PG biosynthetic machines, and yet it contains genetic elements suggestive of both a primary PG biosynthetic machine and a secondary machine [95-97]. In contrast to RodA/MreC/MreD, FtsZ, FtsL, Div1B, and FtsW were deemed essential from our analysis and are likely part of the predominant PG biosynthetic apparatus (Additional file 2). RodA/MreC/MreD may be part of a secondary machine that takes on an important cellular role under environmental stress conditions.

We also identified *mprF* as important for survival at 43°C (16.7 fold depletion). MprF attaches lysine to phosphatidylglycerol in the cell membrane and is involved in cationic antimicrobial peptide resistance [80,98]. Mutants in *mprF* have not previously been shown to be temperature sensitive. Therefore, we constructed an *mprF* deletion strain and confirmed that it exhibited a pronounced growth defect at high temperature (Additional file 1: Figure S6, Figure 6) as it ceased growing completely by mid-log phase. When these cells were plated and grown at 30°C, colonies did not grow, confirming that without a *mprF* gene, high temperatures are lethal.

In contrast to *mprF*, loss of another cell envelope gene, *lyrA,* conferred a growth advantage. Reads due to transposon insertion in this gene were substantially enriched at 43°C compared to 30°C. The growth advantage of a *ΔlyrA* strain compared to wildtype *S. aureus* was confirmed (Figure 6, Additional file 1: Figure S6). Deletion of *lyrA* was previously shown to impart resistance to lysostaphin, an oligopeptide protease that cleaves Gly-Gly bonds in crosslinked PG [88,99,100]. Recent work has implicated LyrA in display of cell surface proteins [101,102], but how its deletion confers increased growth rates at higher temperature is not clear. As an integral membrane protein, LyrA may regulate multiple aspects of cell envelope structure. Perhaps through the loss of interactions with other protein partners, the absence of LyrA induces a pleiotropic cell envelope phenotype capable of withstanding a variety of cell envelope stressors.

### Small colony variant mutants withstand heat stress

Transposon insertion mutants with reads mapping to genes that are part of the aromatic amino acid and menaquinol biosynthetic pathways were significantly enriched at 43°C (Additional file 3, Figure 7). The small colony variant (SCV) phenotype of menaquinone (vitamin K2) mutants in *S. aureus* has been well studied [103-110]. The loss of electron shuttling redox cofactors in the electron transport chain induces a characteristic colony morphology, noted for its slow growth, lack of pigmentation, and pinpoint colony size [107,110-114]. The ensuing reduction in flux through the electron transport chain decreases ATP pools by diminishing respiration, which shifts global metabolism towards fermentation [112,115]. Emergence of SCVs is closely associated with persistent infections [116,117], and SCVs are particularly resistant to many clinically used antibiotics [118,119]. Interestingly, our data revealed a marked growth advantage for menaquinone biosynthetic mutants at 43°C, including *menD, menC, and menF*. Mutations in these same genes have been associated with SCV formation in *S. aureus* clinical isolates, which normally are rapidly outcompeted by the faster growing wild type population [103,107]. Elevated temperature clearly acts as a selective pressure that favors the emergence and propagation of SCVs *in vitro*. Recently, strains with increased resistance to vancomycin were found to emerge in an *in vivo* mouse model independent of vancomycin treatment [120]. Whether the host pyrogenic response induces SCV selection *in vivo,* however, remains an outstanding question.

## Conclusion

In conclusion, we have developed a phage-based method for reproducibly creating high quality, ultra-high density transposon libraries in any *S. aureus* strain transducible by Φ11. We have optimized transposon cassettes and sample preparation procedures to selectively remove transposon-plasmid junctions, and our solutions for reducing background due to these junctions are likely to be useful for other *S. aureus* strains, other bacterial species, and other transposon systems. By multiplexing bar-coded donor constructs, we showed that we can make massive transposon mutant libraries and identify insertions due to each type of donor. In this study, we used the data for the Blunt transposon construct to identify genes important for *S.aureus* survival at high temperatures. Furthermore, we found that SCV mutants are selected for under conditions of heat stress. This system, including the gene expression modulating transposon cassettes, will be useful for future functional genomics analyses aimed at establishing novel strategies for the development of antibacterial agents and garnering insights into the infection biology of *S. aureus.*

## Methods

### Strains and plasmids

Table of strains and plasmids used in this study can be found in Additional file 1 (Additional file 1: Table S3). Original strains and plasmids for the phage-based transposition system were obtained from Merck Research Laboratories [35]. Plasmids were first introduced into the restriction negative *S. aureus* strain RN4220 and then moved into restriction competent HG003 strains by electroporation [121]. Gene deletion strains were made using the *E. coli-S. aureus* shuttle vector pKFC [122]. Stellar competent cells, pUC19, and the In-Fusion cloning kit were purchased from Clontech. Cultures of *S. aureus* were routinely grown in tryptic soy broth (TSB) at the indicated temperature, with erythromycin (5 or 10 μg/mL), chloramphenicol (10 μg/mL), or kanamycin/neomycin (25 μg/mL each) when required. Phage lysates were prepared in TSB top agar and titered as has been previously described [35]. Additional strain construction details can be found in Additional file 1.

### Creation of the transposon library

To create the transposon library, phage lysates were made using the six donor strains harboring plasmids encoding transposon cassettes (pTM239-pTM244) as previously described [35], where one in three progeny phage carries the transposon plasmid. Recipient strains (TM231 and the negative control TM232) were grown to late exponential phase, and resuspended in supplemented glucose minimal media (SGMM) (10 mM glucose, 2 mM MgCl_2_, 3.5 mM CaCl_2_, 0.1% CAS amino acids, 0.5% NaCl, 10 mM MES, pH 6.8). For each transposon construct, phage lysate was mixed with 25 ml recipient strain in SGMM (multiplicity of infection = 5). Solutions were incubated at room temperature overnight. The next day, cells were pelleted and washed three times with an equal volume of TSB before being allowed to recover with shaking for two hours at 30°C. Approximately 10^8^-10^9^ cfu were spread per 150 × 15 mm petri dish containing TSB agar with 5 μg/ml erythromycin to select for transposon insertion mutants. The number of spontaneously erm^R^ colonies was less than 1% in the control strain TM232 that does not express functional transposase. Plates were incubated at 30°C for two days, before being scraped (1-2 million total colonies) and pooled into a single 100 mL suspension. Cells were pelleted and washed three times with an equal volume of TSB, before being resuspended in TSB-glycerol (12.5% v/v), aliquoted, and stored at 80°C. Additional details for the preparation and sequencing of the transposon library can be found in Additional file 1.

### Assessment of next-generation sequencing library composition

A linearized pUC19 vector was made by inverse PCR using primers (Tm179-Tm180) containing 5’-overhangs homologous to the termini of the P5 and P7 Illumina sequences. The 2.6 kb PCR product was agarose gel purified, ligated to an aliquot of the NGS insert library using the Clontech In-Fusion system as directed by the manufacturer, transformed into chemically competent *E. coli* cells, and selected on LB carbenicillin (100 μg/mL) plates. Plasmids were isolated from 2 mL cultures of separate colonies and the DNA inserts sequenced. At least 10 colonies were sequenced to confirm insert sequence diversity and lack of plasmid-transposon junction DNA.

### Next-generation sequencing and data analysis

The DNA concentration was determined using the Quant-IT™ PicoGreen kit from Invitrogen, and the sample was diluted to 10 nM in Buffer EB. Six samples with different indexing barcodes were multiplexed together, and samples were sequenced on a Hi-Seq2000 or Hi-Seq2500 for 100 cycles with 40% ΦX174 spiked in to the sequencing reaction.

Sequencing data were uploaded to the Tufts Galaxy Service [42-44] hosted by TUCF Genomics (http://tucf-genomics.tufts.edu/). The first four base pairs of the 8 bp indexing barcode were deleted since these data were low quality. Multiplexed sample data was separated by the remaining 4 bp of the indexing barcode, and then again by the 3 bp transposon cassette barcode. Read ends were trimmed to leave the 16 bp of genomic sequence immediately flanking the TA dinucleotide insertion site and filtered to keep high-quality reads (>90% of bases with quality score >20). Remaining reads were mapped to the *S. aureus* NCTC8325 genome [44] using Bowtie [123]. Hopcounts (the number of reads that map to a given site in the genome) were determined and these files were downloaded for subsequent statistical analysis. Using custom python scripts modified from ones made available upon request by Eric Rubin (Harvard Medical School), the hopcount reads were mapped to TA sites to create an igv-formatted file. Python scripts (also modified from ones obtained from Eric Rubin) were used to compare the number of reads in the control and experimental conditions using the Mann-Whitney U test [124], correcting for multiple hypothesis testing with the Benjamini-Hochberg procedure [125]. The statistical programming software, R [126], was used to perform the principal component analysis, and the EL-ARTIST essential genes analysis [45] was done in MATLAB [127].

### Growth curves

Growth curves were performed by diluting overnight cultures to an OD_600_ of 0.01 in 25 mL Tryptic Soy Broth. Strains were grown with shaking in a 43°C or 30°C water bath. The OD of the cultures was measured every 10 (43°C) or every 20 (30°C) minutes in an Ultrospec 10 cell density meter until stationary phase.

## Additional files

Additional file 1:
**pdf file contains detailed supplementary data and figures as well as a detailed protocol for preparation of samples for NGS.** All NGS sequencing data will be made available on the publically-accessible Harvard Dataverse Network (http://thedata.harvard.edu/dvn/dv/temp_tnseq_data) in a format in compliance with MINSEQE. Additional file 2:
**excel file contains information pertaining to the essentiality of every gene in** ***S. aureus*** **.** The first three worksheets in this file are named “essential”, “domain essential”, and “nonessential”. They contain the gene locus names for genes classified as essential, domain essential, or nonessential according to the EL-ARTIST analysis as well as the number of TA sites in each gene and the number of sequence reads mapping to each gene. The worksheet named “essential all studies” contains a list of gene loci identified as essential in this work as well as in two other studies [29,33]. The worksheet named “essential by promoter” contains a list of every gene in *S. aureus* and their classification (essential, domain essential, or nonessential) as determined by the EL-ARTIST analysis using the different transposon constructs. Additional file 3:
**excel file contains information regarding the fitness of all genes in** ***S. aureus*** **at different temperatures.** The first worksheet, named “temperatures” contains the hits as determined using the Mann-Whitney U analysis for all temperatures studied. For each hit, the number of TA sites in that gene, the number of sequence reads mapping to that gene for both temperatures, the p-value, and the ratio of the number of reads in the experiment temperature compared to the control temperature are also provided. The worksheet named “43C Essentiality” contains the results of the EL-ARTIST analysis of the 43°C data. Each gene locus and its corresponding classification (essential, domain essential, or nonessential) are provided.

## Abbreviations

MRSA: Methicillin-resistant *Staphylococcus aureus*
PBP: Penicillin-binding protein
NGS: Next-generation sequencing
TraDIS: Transposon-directed insertion-site sequencing
INSeq: Insertion sequencing
Tn-Seq: Transposon sequencing
DNA: Deoxyribonucleic acid
SCV: Small colony variant
ITR: Inverted terminal repeat
PCR: Polymerase chain reaction
PEG: Polyethylene glycol
dsDNA: Double-stranded DNA
CoA: Co-enzyme A
PG: Peptidoglycan
ATPase: Adenosine triphosphatase
ETS: Electron transport system
TSB: Tryptic soy broth
SGMM: Supplemented glucose minimal media
LB: Lysogeny broth
EB: Elution buffer
OD: Optical density
bp: Base pair

## References

[1] Moellering RC (2012). MRSA: the first half century. J Antimicrob Chemother.

[2] Stryjewski ME, Corey GR (2014). Methicillin-resistant Staphylococcus aureus: an evolving pathogen. Clin Infect Dis.

[3] Gould IM, David MZ, Esposito S, Garau J, Lina G, Mazzei T (2012). New insights into meticillin-resistant Staphylococcus aureus (MRSA) pathogenesis, treatment and resistance. Int J Antimicrob Agents.

[4] Antibiotic Resistance Threats in the United States, 2013. [http://www.cdc.gov/drugresistance/threat-report-2013/pdf/ar-threats-2013-508.pdf] 25162160

[5] Roemer T, Schneider T, Pinho MG (2013). Auxiliary factors: a chink in the armor of MRSA resistance to beta-lactam antibiotics. Curr Opin Microbiol.

[6] Berger-Bachi B (1995). Factors affecting methicillin resistance in Staphylococcus aureus. Int J Antimicrob Agents.

[7] Chambers HF (1988). Methicillin-resistant staphylococci. Clin Microbiol Rev.

[8] Utsui Y, Yokota T (1985). Role of an altered penicillin-binding protein in methicillin- and cephem-resistant Staphylococcus aureus. Antimicrob Agents Chemother.

[9] Georgopapadakou NH, Smith SA, Bonner DP (1982). Penicillin-binding proteins in a Staphylococcus aureus strain resistant to specific beta-lactam antibiotics. Antimicrob Agents Chemother.

[10] Brown S, Xia G, Luhachack LG, Campbell J, Meredith TC, Chen C (2012). Methicillin resistance in Staphylococcus aureus requires glycosylated wall teichoic acids. Proc Natl Acad Sci U S A.

[11] Komatsuzawa H, Sugai M, Ohta K, Fujiwara T, Nakashima S, Suzuki J (1997). Cloning and characterization of the fmt gene which affects the methicillin resistance level and autolysis in the presence of triton X-100 in methicillin-resistant Staphylococcus aureus. Antimicrob Agents Chemother.

[12] Berger-Bachi B, Strassle A, Gustafson JE, Kayser FH (1992). Mapping and characterization of multiple chromosomal factors involved in methicillin resistance in Staphylococcus aureus. Antimicrob Agents Chemother.

[13] Campbell J, Singh AK, Santa Maria JP, Kim Y, Brown S, Swoboda JG (2011). Synthetic lethal compound combinations reveal a fundamental connection between wall teichoic acid and peptidoglycan biosyntheses in Staphylococcus aureus. ACS Chem Biol.

[14] Crisostomo MI, Vollmer W, Kharat AS, Inhulsen S, Gehre F, Buckenmaier S (2006). Attenuation of penicillin resistance in a peptidoglycan O-acetyl transferase mutant of Streptococcus pneumoniae. Mol Microbiol.

[15] Dordel J, Kim C, Chung M, Pardos De La Gandara M, Holden MT, Parkhill J (2014). Novel determinants of antibiotic resistance: identification of mutated loci in highly methicillin-resistant subpopulations of methicillin-resistant Staphylococcus aureus. MBio.

[16] Mwangi MM, Kim C, Chung M, Tsai J, Vijayadamodar G, Benitez M (2013). Whole-genome sequencing reveals a link between beta-lactam resistance and synthetases of the alarmone (p)ppGpp in Staphylococcus aureus. Microb Drug Resist.

[17] Qureshi NK, Yin S, Boyle-Vavra S (2014). The role of the Staphylococcal VraTSR regulatory system on vancomycin resistance and vanA operon expression in vancomycin-resistant Staphylococcus aureus. PLoS One.

[18] Boyle-Vavra S, Yin S, Jo DS, Montgomery CP, Daum RS (2013). VraT/YvqF is required for methicillin resistance and activation of the VraSR regulon in Staphylococcus aureus. Antimicrob Agents Chemother.

[19] Meredith TC, Wang H, Beaulieu P, Grundling A, Roemer T (2012). Harnessing the power of transposon mutagenesis for antibacterial target identification and evaluation. Mob Genet Elements.

[20] van Opijnen T, Camilli A (2013). Transposon insertion sequencing: a new tool for systems-level analysis of microorganisms. Nat Rev Microbiol.

[21] Barquist L, Boinett CJ, Cain AK (2013). Approaches to querying bacterial genomes with transposon-insertion sequencing. RNA Biol.

[22] Goodman AL, McNulty NP, Zhao Y, Leip D, Mitra RD, Lozupone CA (2009). Identifying genetic determinants needed to establish a human gut symbiont in its habitat. Cell Host Microbe.

[23] van Opijnen T, Bodi KL, Camilli A (2009). Tn-seq: high-throughput parallel sequencing for fitness and genetic interaction studies in microorganisms. Nat Methods.

[24] Langridge GC, Phan MD, Turner DJ, Perkins TT, Parts L, Haase J (2009). Simultaneous assay of every Salmonella Typhi gene using one million transposon mutants. Genome Res.

[25] Gawronski JD, Wong SM, Giannoukos G, Ward DV, Akerley BJ (2009). Tracking insertion mutants within libraries by deep sequencing and a genome-wide screen for Haemophilus genes required in the lung. Proc Natl Acad Sci U S A.

[26] Kraemer GR, Iandolo JJ (1990). High-Frequency Transformation of Staphylococcus-Aureus by Electroporation. Curr Microbiol.

[27] Schenk S, Laddaga RA (1992). Improved method for electroporation of Staphylococcus aureus. FEMS Microbiol Lett.

[28] Pajunen MI, Pulliainen AT, Finne J, Savilahti H (2005). Generation of transposon insertion mutant libraries for Gram-positive bacteria by electroporation of phage Mu DNA transposition complexes. Microbiology.

[29] Valentino MD, Foulston L, Sadaka A, Kos VN, Villet RA, Santa Maria J (2014). Genes Contributing to Staphylococcus aureus Fitness in Abscess- and Infection-Related Ecologies. MBio.

[30] Santa Maria JP, Sadaka A, Moussa SH, Brown S, Zhang YJ, Rubin EJ (2014). Compound-gene interaction mapping reveals distinct roles for Staphylococcus aureus teichoic acids. Proc Natl Acad Sci U S A.

[31] Bae T, Glass EM, Schneewind O, Missiakas D (2008). Generating a collection of insertion mutations in the Staphylococcus aureus genome using bursa aurealis. Methods Mol Biol.

[32] Fey PD, Endres JL, Yajjala VK, Widhelm TJ, Boissy RJ, Bose JL (2013). A genetic resource for rapid and comprehensive phenotype screening of nonessential Staphylococcus aureus genes. MBio.

[33] Chaudhuri RR, Allen AG, Owen PJ, Shalom G, Stone K, Harrison M (2009). Comprehensive identification of essential Staphylococcus aureus genes using Transposon-Mediated Differential Hybridisation (TMDH). BMC Genomics.

[34] Christiansen MT, Kaas RS, Chaudhuri RR, Holmes MA, Hasman H, Aarestrup FM (2014). Genome-wide high-throughput screening to investigate essential genes involved in methicillin-resistant Staphylococcus aureus Sequence Type 398 survival. PLoS One.

[35] Wang H, Claveau D, Vaillancourt JP, Roemer T, Meredith TC (2011). High-frequency transposition for determining antibacterial mode of action. Nat Chem Biol.

[36] Kilby NJ, Snaith MR, Murray JA (1993). Site-specific recombinases: tools for genome engineering. Trends Genet.

[37] Storici F, Coglievina M, Bruschi CV (1999). A 2-microm DNA-based marker recycling system for multiple gene disruption in the yeast Saccharomyces cerevisiae. Yeast.

[38] Bigot Y, Brillet B, Auge-Gouillou C (2005). Conservation of Palindromic and Mirror Motifs within Inverted Terminal Repeats of mariner-like Elements. J Mol Biol.

[39] Bae T, Banger AK, Wallace A, Glass EM, Aslund F, Schneewind O (2004). Staphylococcus aureus virulence genes identified by bursa aurealis mutagenesis and nematode killing. Proc Natl Acad Sci U S A..

[40] Lis JT, Schleif R (1975). Size Fractionation of Double-Stranded DNA by Precipitation with Polyethylene-Glycol. Nucleic Acids Res.

[41] Georgiou CD, Papapostolou I, Grintzalis K (2009). Protocol for the quantitative assessment of DNA concentration and damage (fragmentation and nicks). Nat Protoc.

[42] Giardine B, Riemer C, Hardison RC, Burhans R, Elnitski L, Shah P (2005). Galaxy: a platform for interactive large-scale genome analysis. Genome Res.

[43] Goecks J, Nekrutenko A, Taylor J (2010). Galaxy: a comprehensive approach for supporting accessible, reproducible, and transparent computational research in the life sciences. Genome Biol.

[44] Blankenberg D, Von Kuster G, Coraor N, Ananda G, Lazarus R, Mangan M (2010). Galaxy: a web-based genome analysis tool for experimentalists. Curr Protoc Mol Biol.

[45] Gillaspy AF, Worrell V, Orvis J, Roe B, Dyer D, Iandolo JJ, Fischetti AF, Novick RP, Ferretti JJ, Portnoy DA, Rood JI (2006). The Staphylococcus aureus NCTC8325 genome. Gram-Positive Pathogens.

[46] Pritchard JR, Chao MC, Abel S, Davis BM, Baranowski C, Zhang YJ (2014). ARTIST: high-resolution genome-wide assessment of fitness using transposon-insertion sequencing. PLoS Genet.

[47] Chao MC, Pritchard JR, Zhang YJ, Rubin EJ, Livny J, Davis BM (2013). High-resolution definition of the Vibrio cholerae essential gene set with hidden Markov model-based analyses of transposon-insertion sequencing data. Nucleic Acids Res.

[48] Krzywinski M, Schein J, Birol I, Connors J, Gascoyne R, Horsman D (2009). Circos: An information aesthetic for comparative genomics. Genome Res.

[49] Kaneda T (1991). Iso-Fatty and Anteiso-Fatty Acids in Bacteria - Biosynthesis, Function, and Taxonomic Significance. Microbiol Rev.

[50] Singh VK, Hattangady DS, Giotis ES, Singh AK, Chamberlain NR, Stuart MK (2008). Insertional inactivation of branched-chain alpha-keto acid dehydrogenase in Staphylococcus aureus leads to decreased branched-chain membrane fatty acid content and increased susceptibility to certain stresses. Appl Environ Microbiol.

[51] Nickel M, Homuth G, Bohnisch C, Mader U, Schweder T (2004). Cold induction of the Bacillus subtilis bkd operon is mediated by increased mRNA stability. Mol Gen Genomics.

[52] Zhu K, Ding X, Julotok M, Wilkinson BJ (2005). Exogenous isoleucine and fatty acid shortening ensure the high content of anteiso-C15:0 fatty acid required for low-temperature growth of Listeria monocytogenes. Appl Environ Microbiol.

[53] Duman R, Ishikawa S, Celik I, Strahl H, Ogasawara N, Troc P (2013). Structural and genetic analyses reveal the protein SepF as a new membrane anchor for the Z ring. Proc Natl Acad Sci U S A.

[54] Szurmant H, Nelson K, Kim EJ, Perego M, Hoch JA (2005). YycH regulates the activity of the essential YycFG two-component system in Bacillus subtilis. J Bacteriol.

[55] Turck M, Bierbaum G (2012). Purification and Activity Testing of the Full-Length YycFGHI Proteins of Staphylococcus aureus. PLoS One.

[56] Szurmant H, Mohan MA, Imus PM, Hoch JA (2007). YycH and YycI interact to regulate the essential YycFG two-component system in Bacillus subtilis. J Bacteriol.

[57] Dubrac S, Boneca IG, Poupel O, Msadek T (2007). New insights into the WalK/WalR (YycG/YycF) essential signal transduction pathway reveal a major role in controlling cell wall metabolism and biofilm formation in Staphylococcus aureus. J Bacteriol.

[58] Ohta T, Saito K, Kuroda M, Honda K, Hirata H, Hayashi H (1994). Molecular cloning of two new heat shock genes related to the hsp70 genes in Staphylococcus aureus. J Bacteriol.

[59] Chastanet A, Fert J, Msadek T (2003). Comparative genomics reveal novel heat shock regulatory mechanisms in Staphylococcus aureus and other Gram-positive bacteria. Mol Microbiol.

[60] Chatterjee I, Becker P, Grundmeier M, Bischoff M, Somerville GA, Peters G (2005). Staphylococcus aureus ClpC is required for stress resistance, aconitase activity, growth recovery, and death. J Bacteriol.

[61] Derre I, Rapoport G, Msadek T (1999). CtsR, a novel regulator of stress and heat shock response, controls clp and molecular chaperone gene expression in gram-positive bacteria. Mol Microbiol.

[62] Wozniak DJ, Tiwari KB, Soufan R, Jayaswal RK (2012). The mcsB gene of the clpC operon is required for stress tolerance and virulence in Staphylococcus aureus. Microbiology.

[63] Zylicz M, Ang D, Liberek K, Georgopoulos C (1989). Initiation of lambda DNA replication with purified host- and bacteriophage-encoded proteins: the role of the dnaK, dnaJ and grpE heat shock proteins. EMBO J.

[64] Kozarich JW, Strominger JL (1978). A membrane enzyme from Staphylococcus aureus which catalyzes transpeptidase, carboxypeptidase, and penicillinase activities. J Biol Chem.

[65] Pinho MG, de Lencastre H, Tomasz A (2000). Cloning, characterization, and inactivation of the gene pbpC, encoding penicillin-binding protein 3 of Staphylococcus aureus. J Bacteriol.

[66] Qiao Y, Lebar MD, Schirner K, Schaefer K, Tsukamoto H, Kahne D (2014). Detection of lipid-linked peptidoglycan precursors by exploiting an unexpected transpeptidase reaction. J Am Chem Soc.

[67] Blake KL, O'Neill AJ, Mengin-Lecreulx D, Henderson PJ, Bostock JM, Dunsmore CJ (2009). The nature of Staphylococcus aureus MurA and MurZ and approaches for detection of peptidoglycan biosynthesis inhibitors. Mol Microbiol.

[68] Kullik I, Jenni R, Berger-Bachi B (1998). Sequence of the putative alanine racemase operon in Staphylococcus aureus: insertional interruption of this operon reduces D-alanine substitution of lipoteichoic acid and autolysis. Gene.

[69] Heidrich C, Templin MF, Ursinus A, Merdanovic M, Berger J, Schwarz H (2001). Involvement of N-acetylmuramyl-L-alanine amidases in cell separation and antibiotic-induced autolysis of Escherichia coli. Mol Microbiol.

[70] Muchova K, Chromikova Z, Barak I (2013). Control of Bacillus subtilis cell shape by RodZ. Environ Microbiol.

[71] Garner EC, Bernard R, Wang W, Zhuang X, Rudner DZ, Mitchison T (2011). Coupled, circumferential motions of the cell wall synthesis machinery and MreB filaments in B. subtilis. Science.

[72] Dominguez-Escobar J, Chastanet A, Crevenna AH, Fromion V, Wedlich-Soldner R, Carballido-Lopez R (2011). Processive movement of MreB-associated cell wall biosynthetic complexes in bacteria. Science.

[73] Henriques AO, Glaser P, Piggot PJ, Moran CP (1998). Control of cell shape and elongation by the rodA gene in Bacillus subtilis. Mol Microbiol.

[74] Steele VR, Bottomley AL, Garcia-Lara J, Kasturiarachchi J, Foster SJ (2011). Multiple essential roles for EzrA in cell division of Staphylococcus aureus. Mol Microbiol.

[75] Lithgow JK, Ingham E, Foster SJ (2004). Role of the hprT-ftsH locus in Staphylococcus aureus. Microbiology.

[76] Claessen D, Emmins R, Hamoen LW, Daniel RA, Errington J, Edwards DH (2008). Control of the cell elongation-division cycle by shuttling of PBP1 protein in Bacillus subtilis. Mol Microbiol.

[77] Liu G, Draper GC, Donachie WD (1998). FtsK is a bifunctional protein involved in cell division and chromosome localization in Escherichia coli. Mol Microbiol.

[78] Chan YG, Frankel MB, Dengler V, Schneewind O, Missiakas D (2013). Staphylococcus aureus mutants lacking the LytR-CpsA-Psr family of enzymes release cell wall teichoic acids into the extracellular medium. J Bacteriol.

[79] Atilano ML, Pereira PM, Yates J, Reed P, Veiga H, Pinho MG (2010). Teichoic acids are temporal and spatial regulators of peptidoglycan cross-linking in Staphylococcus aureus. Proc Natl Acad Sci U S A.

[80] Oku Y, Kurokawa K, Ichihashi N, Sekimizu K (2004). Characterization of the Staphylococcus aureus mprF gene, involved in lysinylation of phosphatidylglycerol. Microbiology.

[81] Caspi R, Altman T, Dale JM, Dreher K, Fulcher CA, Gilham F (2010). The MetaCyc database of metabolic pathways and enzymes and the BioCyc collection of pathway/genome databases. Nucleic Acids Res.

[82] Bentley R, Meganathan R (1982). Biosynthesis of vitamin K (menaquinone) in bacteria. Microbiol Rev.

[83] Nowicka B, Kruk J (2010). Occurrence, biosynthesis and function of isoprenoid quinones. Biochim Biophys Acta.

[84] Hansson M, Hederstedt L (1992). Cloning and characterization of the Bacillus subtilis hemEHY gene cluster, which encodes protoheme IX biosynthetic enzymes. J Bacteriol.

[85] McNamara PJ, Proctor RA (2000). Staphylococcus aureus small colony variants, electron transport and persistent infections. Int J Antimicrob Agents.

[86] Baumert N, von Eiff C, Schaaff F, Peters G, Proctor RA, Sahl HG (2002). Physiology and antibiotic susceptibility of Staphylococcus aureus small colony variants. Microb Drug Resist.

[87] Margot P, Mauel C, Karamata D (1994). The gene of the N-acetylglucosaminidase, a Bacillus subtilis 168 cell wall hydrolase not involved in vegetative cell autolysis. Mol Microbiol.

[88] Grundling A, Missiakas DM, Schneewind O (2006). Staphylococcus aureus mutants with increased lysostaphin resistance. J Bacteriol.

[89] Bae T, Baba T, Hiramatsu K, Schneewind O (2006). Prophages of Staphylococcus aureus Newman and their contribution to virulence. Mol Microbiol.

[90] Goodman AL, Wu M, Gordon JI (2011). Identifying microbial fitness determinants by insertion sequencing using genome-wide transposon mutant libraries. Nat Protoc.

[91] Anderson KL, Roberts C, Disz T, Vonstein V, Hwang K, Overbeek R (2006). Characterization of the Staphylococcus aureus heat shock, cold shock, stringent, and SOS responses and their effects on log-phase mRNA turnover. J Bacteriol.

[92] Costa CS, Anton DN (1993). Round-cell mutants of Salmonella typhimurium produced by transposition mutagenesis: lethality of rodA and mre mutations. Mol Gen Genet.

[93] Sham LT, Tsui HC, Land AD, Barendt SM, Winkler ME (2012). Recent advances in pneumococcal peptidoglycan biosynthesis suggest new vaccine and antimicrobial targets. Curr Opin Microbiol.

[94] Zapun A, Vernet T, Pinho MG (2008). The different shapes of cocci. FEMS Microbiol Rev.

[95] Pinho MG, Errington J (2003). Dispersed mode of Staphylococcus aureus cell wall synthesis in the absence of the division machinery. Mol Microbiol.

[96] Pinho MG, Kjos M, Veening JW (2013). How to get (a)round: mechanisms controlling growth and division of coccoid bacteria. Nat Rev Microbiol.

[97] Margolin W (2009). Sculpting the bacterial cell. Curr Biol.

[98] Nishi H, Komatsuzawa H, Fujiwara T, McCallum N, Sugai M (2004). Reduced content of lysyl-phosphatidylglycerol in the cytoplasmic membrane affects susceptibility to moenomycin, as well as vancomycin, gentamicin, and antimicrobial peptides, in Staphylococcus aureus. Antimicrob Agents Chemother.

[99] Grundling A, Schneewind O (2006). Cross-linked peptidoglycan mediates lysostaphin binding to the cell wall envelope of Staphylococcus aureus. J Bacteriol.

[100] Schindler CA, Schuhardt VT (1964). Lysostaphin: A New Bacteriolytic Agent for the Staphylococcus. Proc Natl Acad Sci U S A.

[101] Frankel MB, Wojcik BM, DeDent AC, Missiakas DM, Schneewind O (2010). ABI domain-containing proteins contribute to surface protein display and cell division in Staphylococcus aureus. Mol Microbiol.

[102] Pei J, Mitchell DA, Dixon JE, Grishin NV (2011). Expansion of type II CAAX proteases reveals evolutionary origin of gamma-secretase subunit APH-1. J Mol Biol.

[103] Dean MA, Olsen RJ, Long SW, Rosato AE, Musser JM (2014). Identification of point mutations in clinical Staphylococcus aureus strains that produce small-colony variants auxotrophic for menadione. Infect Immun.

[104] Biswas L, Biswas R, Schlag M, Bertram R, Gotz F (2009). Small-colony variant selection as a survival strategy for Staphylococcus aureus in the presence of Pseudomonas aeruginosa. Appl Environ Microbiol.

[105] Mayfield JA, Hammer ND, Kurker RC, Chen TK, Ojha S, Skaar EP (2013). The chlorite dismutase (HemQ) from Staphylococcus aureus has a redox-sensitive heme and is associated with the small colony variant phenotype. J Biol Chem.

[106] Onyango LA, Hugh Dunstan R, Roberts TK, Macdonald MM, Gottfries J (2013). Phenotypic variants of staphylococci and their underlying population distributions following exposure to stress. PLoS One.

[107] von Eiff C, Heilmann C, Proctor RA, Woltz C, Peters G, Gotz F (1997). A site-directed Staphylococcus aureus hemB mutant is a small-colony variant which persists intracellularly. J Bacteriol.

[108] von Eiff C, McNamara P, Becker K, Bates D, Lei XH, Ziman M (2006). Phenotype microarray profiling of Staphylococcus aureus menD and hemB mutants with the small-colony-variant phenotype. J Bacteriol.

[109] Jensen J (1957). Biosynthesis of hematin compounds in a hemin requiring strain of Micrococcus pyogenes var. aureus. I. The significance of coenzyme A for the terminal synthesis of catalase. J Bacteriol.

[110] Proctor RA, von Eiff C, Kahl BC, Becker K, McNamara P, Herrmann M (2006). Small colony variants: a pathogenic form of bacteria that facilitates persistent and recurrent infections. Nat Rev Microbiol.

[111] Proctor RA, Balwit JM, Vesga O (1994). Variant subpopulations of Staphylococcus aureus as cause of persistent and recurrent infections. Infect Agents Dis.

[112] Proctor RA, van Langevelde P, Kristjansson M, Maslow JN, Arbeit RD (1995). Persistent and relapsing infections associated with small-colony variants of Staphylococcus aureus. Clin Infect Dis.

[113] Bates DM, von Eiff C, McNamara PJ, Peters G, Yeaman MR, Bayer AS (2003). Staphylococcus aureus menD and hemB mutants are as infective as the parent strains, but the menadione biosynthetic mutant persists within the kidney. J Infect Dis.

[114] Clements MO, Watson SP, Poole RK, Foster SJ (1999). CtaA of Staphylococcus aureus is required for starvation survival, recovery, and cytochrome biosynthesis. J Bacteriol.

[115] Kohler C, von Eiff C, Peters G, Proctor RA, Hecker M, Engelmann S (2003). Physiological characterization of a heme-deficient mutant of Staphylococcus aureus by a proteomic approach. J Bacteriol.

[116] Wolter DJ, Emerson JC, McNamara S, Buccat AM, Qin X, Cochrane E (2013). Staphylococcus aureus small-colony variants are independently associated with worse lung disease in children with cystic fibrosis. Clin Infect Dis.

[117] Tan NC, Cooksley CM, Roscioli E, Drilling AJ, Douglas R, Wormald PJ (2014). Small-colony variants and phenotype switching of intracellular Staphylococcus aureus in chronic rhinosinusitis. Allergy.

[118] Lechner S, Lewis K, Bertram R (2012). Staphylococcus aureus persisters tolerant to bactericidal antibiotics. J Mol Microbiol Biotechnol.

[119] Chuard C, Vaudaux PE, Proctor RA, Lew DP (1997). Decreased susceptibility to antibiotic killing of a stable small colony variant of Staphylococcus aureus in fluid phase and on fibronectin-coated surfaces. J Antimicrob Chemother.

[120] Koch G, Yepes A, Forstner KU, Wermser C, Stengel ST, Modamio J (2014). Evolution of Resistance to a Last-Resort Antibiotic in Staphylococcus aureus via Bacterial Competition. Cell.

[121] Lee JC (1995). Electrotransformation of Staphylococci. Methods Mol Biol.

[122] Kato F, Sugai M (2011). A simple method of markerless gene deletion in Staphylococcus aureus. J Microbiol Methods.

[123] Langmead B, Trapnell C, Pop M, Salzberg SL (2009). Ultrafast and memory-efficient alignment of short DNA sequences to the human genome. Genome Biol.

[124] Mann HB, Whitney DR (1947). On a Test of Whether One of 2 Random Variables Is Stochastically Larger Than the Other. Ann Math Stat.

[125] Benjamini Y, Hochberg Y (1995). Controlling the False Discovery Rate - a Practical and Powerful Approach to Multiple Testing. J Royal Statistical Soc Series B-Methodol.

[126] R: A language and environment for statistical computing. [http://www.R-project.org/]

[127] MATLAB and Statistics Toolbox Release R2014a edition. Natick, MA: The Mathworks, Inc; 2014.

