# Supplementary material for: A new platform for ultra-high density Staphylococcus aureus transposon libraries
Source: BMC Genomics. 2015 Mar 29;16(1):252. doi: 10.1186/s12864-015-1361-3 (PMC4389836; doi:10.1186/s12864-015-1361-3)
Supplement: Additional file 1: — pdf file contains detailed supplementary data and figures as well as a detailed protocol for preparation of samples for NGS. All NGS sequencing data will be made available on the publically-accessible Harvard Dataverse Network (http://thedata.harvard.edu/dvn/dv/temp_tnseq_data) in a format in compliance with MINSEQE. [file 12864_2015_1361_MOESM1_ESM.pdf]

ADDITIONAL FILE 1

ADDITIONAL METHODS

Strain construction

To make the plasmids pORF5 Tnp+ and pORF5 Tnp-, which express an active or truncated C9 HMAR transposase along with the c*I*-like repressor of Φ11, the ORF5 gene with the presumptive promoter region was PCR amplified from Φ11 using primers Tm1-Tm2 (Table S4). The PCR product was digested with HindIII/EcoRI, and ligated with T4 DNA ligase into either pTM378 or pTM381 that had previously been cut with HindIII/MfeI. The purified ligation mix was directly electroporated into RN4220, and plated at 30°C to select for kanamycin resistant colonies. Plasmids were purified and verified by DNA sequencing to generate RN4220 recipient strains (TM174 and TM175). Strain HG003 was then transformed with the plasmids to generate strains TM176 and TM177, respectively.

The transposon donor plasmid series (pTM239-244), containing the MmeI-ITR element, P7 Illumina primer annealing region, flanking NotI sites, and outward facing regulatory elements with unique 3-bp DNA bar codes was constructed from pTM402. The ITR1 region was first replaced by digesting pTM402 with SphI/ScaI, and then ligating the Tm138 synthetic DNA fragment (gBlock from IDT) containing an optimized ITR using the In-Fusion kit. The ITR2 of the resulting plasmid was likewise replaced with the gBlock DNA fragment Tm140 post digestion with BamHI/MluI, encoding a MmeI modified ITR. The NotI-P7-NNN (3-bp randomized) was introduced into the transposon backbone upstream of ITR2 by inverse PCR using primers Tm162-Tm166, digested with MluI, and circularized by ligation. A 6-member set of stock plasmids with unique NNN barcodes was assembled, with a single construct being assigned as Blunt due to the lack of outward facing regulatory elements (pTM239). The promoter constructs (pTM240-242 with P_{pen}, P_{cap}, and P_{tuf}, respectively) were assembled by amplifying each promoter element and ligating into a stock plasmid using the BspHI/AscI restriction sites as has been described [1]. The P_{erm} construct (pTM243) was made by removing the endogenous

transcriptional terminator of the erythromycin resistance gene by inverse PCR (primers Tm167-170) of a stock plasmid, 5'-phosphorylation with T4 polynucleotide kinase, and self-ligation. Finally, a construct containing dual outward facing promoters (pTM244) was made in two steps by both ligating the P_{pen} promoter element and removing the transcriptional terminator as described.

The Φ11-FRT phage variant, which does not express a functional integrase gene and lacks the *attP* site, was created by sequentially exchanging each of the *attL/attR* attachment sites of the Φ11 prophage in HG003 with the FRT site specific recombination sequence from *Saccharomyces cerevesiae* [2, 3]. The allelic exchange vectors pTM204*attLint* and pTM204*attR* were constructed by 3-piece overlap assembly of 1-kb DNA regions flanking the respective *att* site with EcoRI/HindIII digested pKFC using the In-Fusion Kit from Clontech. The FRT DNA sequence was introduced during PCR into the 5'-tail of the P2-P3 primers (Table S3). Allelic exchange at both sites in strain TM222 was confirmed by PCR using flanking primers. The FLP expressing vector pTM195 was next constructed by ligating the FLP recombinase gene, amplified with primers Tm79-Tm80 from pCP20, to the pLI50-Ppen *E. coli-S. aureus* shuttle vector digested with BamHI/AscI. The *att*-defective prophage of TM222 was induced by electroporation of pTM195, which constitutively expresses the FLP recombinase, and supplementation of outgrowth media with 1 µg/mL of mitomycin C. After 3 hours of growth at 30°C, cells were removed by centrifugation and the serial dilutions of supernatant were added to an RN4220 top agar overlay. Discrete plaques were isolated, and the expected *attP-int::*FRT locus was confirmed by sequencing. To selectively excise the Φ11 prophage in HG003, TM222 was likewise utilized except an aliquot of the pTM195 electroporation cell suspension was directly plated on TSB agar (Cm 10 µg/mL) without prior mitomycin C treatment. Colonies containing plasmid were then subcultured overnight in 10 mL of TSB to allow for Φ11 prophage excision and plasmid loss, before being plated to single colonies. Colonies were replica plated to check for Cm sensitivity, indicating loss of pTM195, and sensitivity to Φ11 infection. The

Φ11::FRT locus of one colony with the expected phenotype, TM226, was confirmed by DNA sequencing.

Gene deletion and replacement with the kan^R gene were done as previously described with few modifications [4]. Briefly, primers lyrA1-6 or mprF1-6 were used to amplify 1000bp upstream and 1000 bp downstream of the target gene to be deleted as well as the kan^R fragments. Equimolar concentrations of the three fragments (-1000, kan^R, and +1000) were spliced by PCR, digested with BamHI-HF and SalI-HF (NEB) and ligated into the pKFC plasmid digested with the same restriction enzymes. RN4220 was transformed with each plasmid and deletion/replacement of the target gene with kan^R was performed by single crossover integration of the plasmid at 42°C and curing of the plasmid at 30°C. Deletion/replacements were verified by PCR and genomic DNA sequencing and subsequently transduced into *S. aureus* HG003 using phage Φ85.

Preparation and sequencing of transposon library

Unless otherwise described, reactions are mixed and incubated in 1.5 mL Eppendorf LoBind tubes. DNA was stored at -20°C overnight, and at -80°C long term. At least 10 μg of high molecular weight genomic DNA was purified using the GES protocol [5]. 10 μg of genomic DNA was digested with 50-100 U NotI in a 600 μl reaction in NEB Buffer #3 supplemented with BSA. The reaction was vortexed gently, mixed by inversion, spun down, and incubated at 37°C for seven hours (mixing and spinning down once halfway through). NotI was inactivated at 70°C for 20 minutes, and cooled to room temperature (5 minutes).

Next, the transposon-plasmid junctions were removed through a size-selective precipitation [6, 7]. The 4x Precipitation buffer contains 32% PEG8000, 2.2M NaCl (autoclaved), 40mM Na_2PO_4 or K_2PO_4 pH 7.5 (autoclaved), and brought to volume with autoclaved ddH_2O. 200μl of 4x precipitation buffer was added to the 600μl digest reaction and vortexed and inverted (not pipetted) to mix. This reaction was incubated in an ice water bath in a 4°C cold room for 12-16 hours. After incubation, the reaction was spun down in a tabletop centrifuge at

maximum speed for 20 minutes at 4°C. Then, the supernatant was removed, and the precipitated DNA was washed once with cold 1x Precipitation buffer, spinning down the DNA at maximum speed for 10 minutes at 4°C. To further purify the DNA, pellets were washed twice with room temperature 70% ethanol, spinning down the DNA at maximum speed for 5 minutes at room temperature between washes. The final DNA pellet was dried and resuspended in 50-100µl standard elution buffer (10mM Tris, pH 8.5) by pipetting.

A PCR check was performed on one sample (undigested and NotI digested) to confirm that the DNA was sufficiently digested. TM196 and TM198 are experimental primers that anneal to the transposon on either side of a NotI site. TM58 and TM59 are control primers and anneal to a genomic fragment inside the *tarK* gene. Seven reactions were set up for each primer set for both undigested and digested DNA. The 25µl PCR reaction consisted of 2.5µl 10x KOD Hot Start Buffer, 5 µl 5x CES mix, 1.5µl 25mM $MgSO_4$, 2.5 µl 2mM dNTPs, 0.25 µl of each primer (experimental or control), 0.25 µl KOD Hot Start Polymerase, 25 ng of DNA (digested or undigested), and autoclaved ddH_2O to bring the reaction to 25 µl. Reactions were incubated in a thermocycler at 95°C for 2 minutes; with 30 cycles of 95°C for 20 seconds, 55°C for 20 seconds, and 70°C for 30 seconds; and 12°C for storage. CES buffer consists of 2.7M betaine, 6.7mM DTT, 6.7% DMSO, and 55µg/ml BSA [8]. Beginning at the end of 9 cycles, a tube from each category of reaction (undigested with experimental primers, digested with experimental primers, undigested with control primers, digested with control primers) was removed from the thermocycler every three cycles and quenched with DNA gel loading buffer supplemented with 0.1% SDS. Samples were stored at -20°C until all cycles were completed. The final tubes were removed at the end of the 27th cycle. These reactions were run on a 1% agarose gel in TAE buffer. In a sample deemed sufficiently digested, a six cycle product detection threshold difference between the undigested and digested samples when using the experimental primers (785 bp) in comparison to the control primers (1298 bp) was required (Figure S3).

Once we confirmed that the DNA had been sufficiently digested, the biotinylated adaptors were ligated. 50 µM of annealed adaptors were prepared by mixing 15 µl of 100 µM TM214 and 15 µl of 100 µM TM215 with 1.5 µl 1M NaCl. This reaction was incubated in a thermocycler at 95°C for 5 minutes followed by cooling to 4°C at a rate of 0.1°C/second. Each 150µl ligation reaction consisted of 15 µl of 10x T4 ligase buffer, 8 µg digested DNA in 100 µl of ddH_2O, 3 µl of T4 ligase enzyme, 4.5 µl of annealed adaptors diluted 1:10 to 5 µM in ice cold 1x T4 ligase buffer, and 27.5 µl autoclaved ddH_2O. Reactions were incubated at 16°C overnight. Another size-selective precipitation was performed to remove un-ligated adaptors from genomic DNA. 50 µl of the same 4x PEG solution described above was added to the ligation reaction, and the tube mixed by vortexing, spun down, and incubated at 4°C or in the cold room for 12-16 hours. After incubation, the DNA is washed and dried in the same manner as described above after the NotI digest.

Next, transposon-genome junctions were captured by digesting the DNA with MmeI which digests 20 bp 3' from its asymmetric recognition site near the end of the transposon ITR. The M12 oligonucleotides were first annealed together as described above to create 50 µM double-stranded DNA. MmeI digests take place in 200µl reactions and require at least 5 µg of DNA from the previous step diluted to 50 µl in autoclaved ddH_2O. MmeI reactions consist of 20 µl 10x NEB CutSmart Buffer, 0.8 µl 32 mM SAM, 2 µl 50 µM annealed M12 oligos, 50 µl DNA, 2 µl MmeI enzyme, and 125.2 µl autoclaved ddH_2O. The reactions were incubated at 37°C for two hours.

MmeI-digested DNA was ligated to streptavidin dynabeads via the biotinylated adaptor. This required three buffers. The 2x BandW Buffer consists of 2 M NaCl, 10 mM Tris, and 1 mM EDTA pH 7.5 with concentrated HCl [9]. LoTE Buffer consists of 3 mM Tris, 0.2 mM EDTA pH 7.5 with concentrated HCl [9]. LoTE+Tween Buffer is the same as LoTE buffer, but supplemented with 0.05% Tween 20. 200 µl of 2x BandW buffer was added to each sample. 32 µl/sample of Dynabeads® M-280 Streptavidin beads was added to a LoBind tube and placed in

the magnetic particle collector (MPC). Then, the supernatant was removed, and the beads were washed three times with 1 mL of 1x BandW buffer. Finally, the sample was resuspended in 32 µl/sample of 1x BandW buffer, and 32 µl of beads were added to each diluted MmeI digest sample. These were incubated at room temperature for one hour, resuspending by tapping and inversion every 10-15 minutes. At this point, all biotinylated DNA should be bound to the beads, so the beads were collected using the MPC, washed once with LoTE+Tween and twice with LoTE (no Tween). Finally, the beads were resuspended in 100µl of LoTE, and transferred to a PCR tube. Beads can be stored in LoTE buffer at 4°C overnight.

With one end of the DNA attached to beads, the other Illumina adaptors with index barcodes were ligated to the other end. We used six adaptors (LIB_AdaptT_1_long, LIB_AdaptB_1_long, LIB_AdaptT_2_long, LIB_AdaptB_2_long, LIB_AdaptT_3_long, LIB_AdaptB_3_long, LIB_AdaptT_4_long, LIB_AdaptB_4_long, LIB_AdaptT_5_long, LIB_AdaptB_5_long, LIB_AdaptT_6_long, LIB_AdaptB_6_long) annealed to each other (T to B) as described above. We would like to note that neither the barcodes in these adaptors nor the transposon construct-specific barcodes use an error-correcting barcode sequence [10, 11]. Without error correcting barcodes, it is possible that sequences could be mis-assigned to the wrong sample or wrong transposon construct due to errors in sequencing. However, because we only used sequences with a high quality score in our analysis, we assume that the fraction of mis-assigned sequences is negligible. These double-stranded adaptors were diluted tenfold to 5 µM in ice cold T4 ligase buffer. The ligation mix consisted of 16.4 µl ddH_2O and 2 µl 10x T4 ligase buffer per sample. Beads were collected in the MPC and resuspended in 18.4 µl ligation mix. 0.6 µl of the double-stranded adaptor is added to each tube, a different adaptor to each tube. Then 1 µl of T4 ligase is added to each tube. The reaction is mixed by pipetting, and the reactions were incubated for 6-7 hours at 16°C in a thermocycler, mixing by tapping and pipetting every 15 minutes. After incubation, the beads were washed once with 150 µl

LoTE+Tween and twice with 150 μl LoTE. Again, beads can be stored in LoTE buffer at 4°C overnight

The final PCR reaction amplifies the transposon-genome junction off of the beads and adds the adaptor sequences required for Illumina sequencing. Beads were collected in the MPC and resuspended in 50 μl of the final PCR reaction mix. This 50 μl PCR reaction consists of 5 μl 10x KOD buffer, 3 μl 25 mM $MgSO_4$, 5 μl 2 mM dNTPs, 35.5 μl autoclaved ddH_2O, 0.5 μl 100μM TM199, 0.5 μl 100 μM LIB-PCR_3, and 0.5 μl KOD Hot Start Polymerase per sample. The reaction was incubated in the thermocycler using the following program: 95°C for 2 minutes; 15-18 cycles of 95°C for 20 seconds, 60°C for 20 seconds, 72°C for 20 seconds. The beads were collected, and the supernatant was transferred to new tubes where DNA loading buffer was added, and the samples were run on a 2% agarose gel in TAE at 130V for 20 minutes. Bands of the expected size (161 bp) were extracted using the Qiagen gel extraction kit with a few minor modifications. The gel fragments were dissolved in Buffer QG at room temperature to prevent dissociation of the short strands of DNA. After the final Buffer PE wash, excess buffer was removed from the column by pipette. The DNA was eluted in 30 μl Buffer EB, and DNA concentration was determined using the Quant-IT^{TM} PicoGreen kit from Invitrogen. The final Tn-Seq sample was diluted to 10 nM in Buffer EB, and six samples with different barcodes were typically multiplexed together in a single lane. Samples were sequenced on a Hi-Seq2000 or Hi-Seq2500 for 100 cycles with 40% ΦX174 spiked in to the sequencing reaction.

ADDITIONAL FIGURES

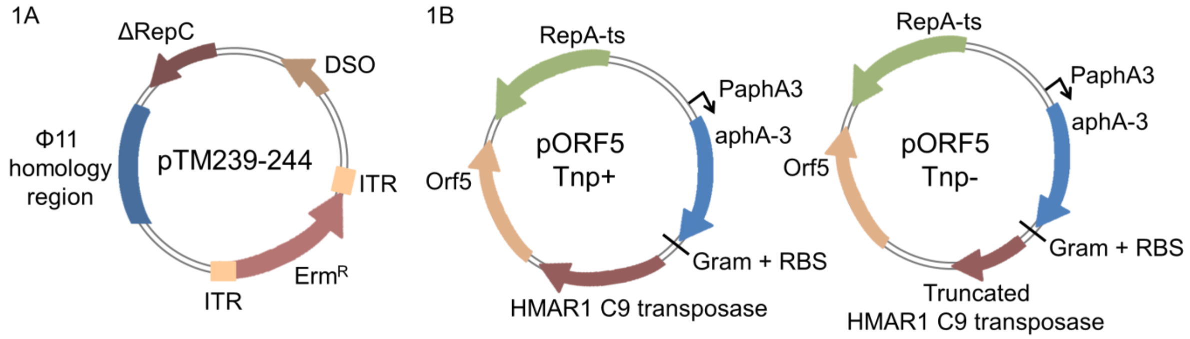

Figure S1. Plasmid Maps. (*A*) The transposon donor plasmid has a non-functional replication initiation protein (*ΔRepC*) but the double stranded origin (DSO) is present to allow replication *in trans* in *repC*$^{+}$ expressing hosts. The Φ11 homology region promotes recombination and packaging as head-to-tail concatemers of donor plasmids. (*B*) Plasmid maps of C9 HMAR1 transposase expressing (pORF5- Tnp+) and control (pORF5-Tnp-) constructs. Elements include the ORF5 gene under control of its own promoter to prevent replication of wt Φ11, the temperature sensitive allele of the RepA Gram positive replication origin, and kanamycin resistance gene (*aphA-3*).

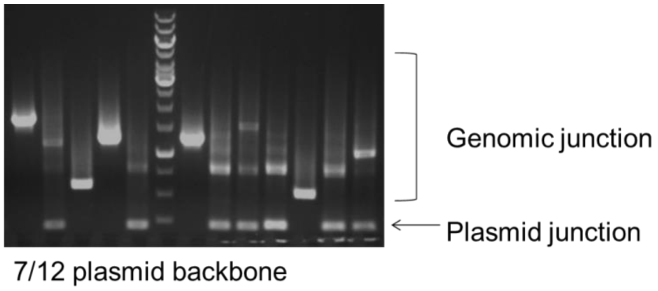

Figure S2. Plasmid-transposon junction content with MmeI-modified ITR site. Upon incorporation of an *Mme*I in ITR2, the fraction of transposon insertion mutants harboring plasmid-transposon junctions increased from 3/12 (not shown) to 7/12.

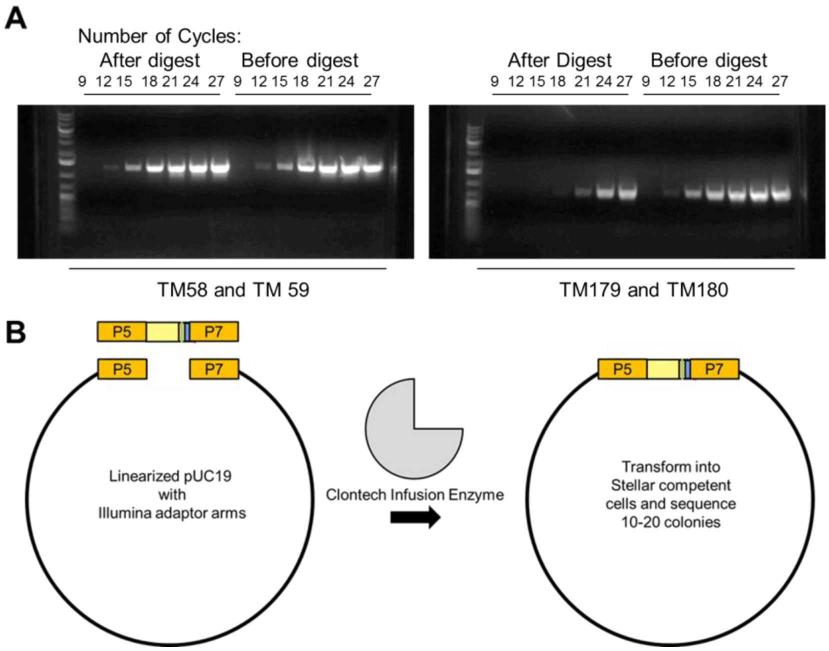

Figure S3. PCR and NGS library diversity analysis. (*A*) The amount of transposon-plasmid junction was quantified by removing aliquots of each PCR reaction at the indicated cycle number and analyzing by agarose gel electrophoresis. The reduction in the detection threshold cycle number for the transposon-plasmid junction PCR using primers TM179 and TM180 had to be reduced by at least 6 cycles after *Not*I digestion in order for the sample to be further processed. Amplification of a genomic locus using control primers TM58 and TM59 was used as control (top panel). (*B*) NGS library was cloned into a pUC19 vector with ends homologous to the P5 and P7 Illumina primers. Individual colonies were sequenced to confirm insert diversity before being submitted for NGS.

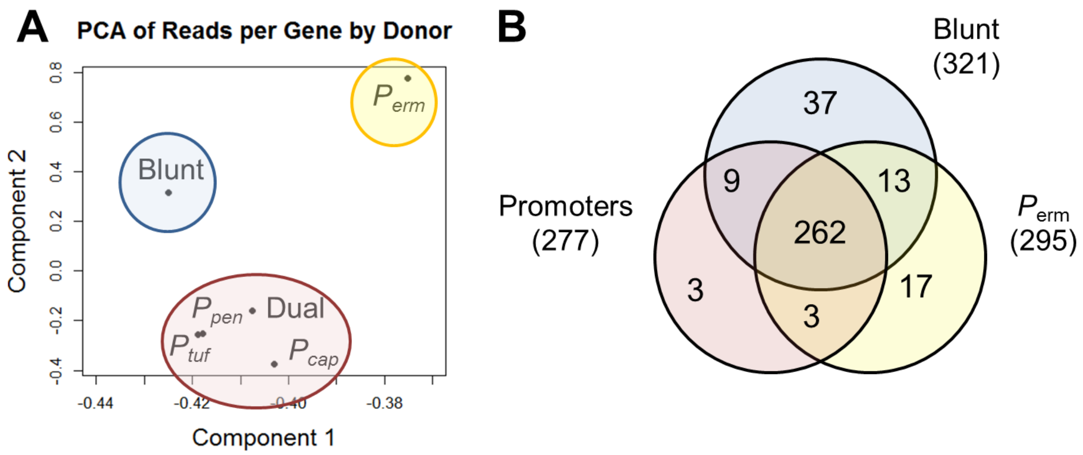

Figure S4. Comparison of transposon donor constructs. *(A)* Principal component analysis was used to compare the number of reads mapping to each gene for each of the six transposon donor constructs. Those transposon constructs with outward-facing promoters clustered together. Data for these constructs were combined and analyzed separately. (*B)* Venn diagram showing the numbers of essential genes for each category of donor construct as calculated using EL-ARTIST [12].

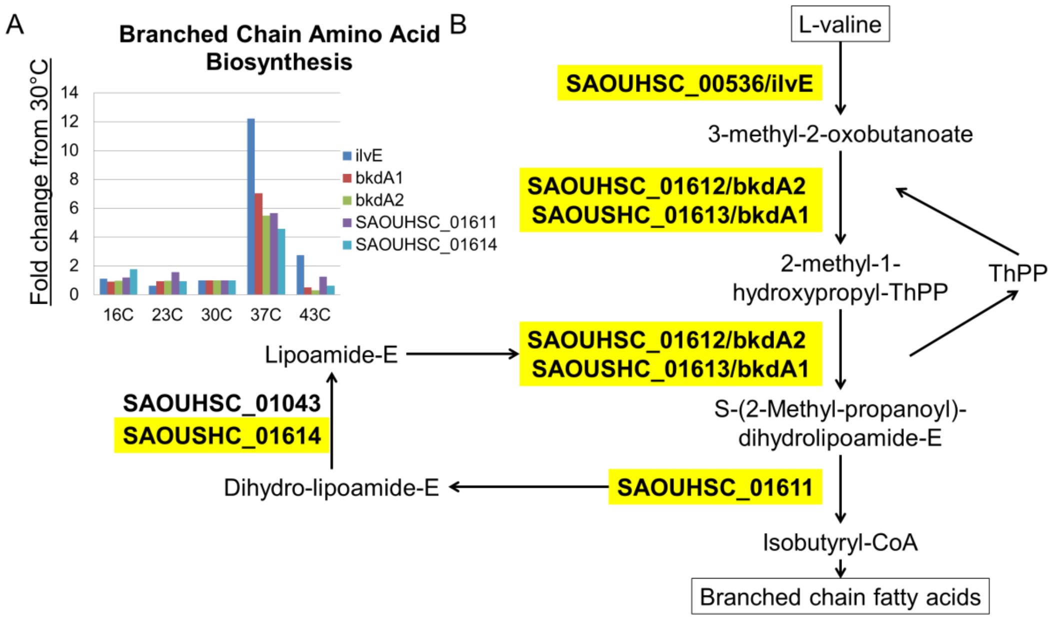

Figure S5. Enrichment of transposon insertions in the branched chain amino acid degradation pathway. (*A*) The number of reads in each gene per 5 million reads was normalized to the read count at 30°C and plotted for each temperature. (*B*) Four genes in the branched chain fatty acid pathway were found to have a statistically-significant increase in the number of reads and SAOUHSC_01614 was found to have a non-statistically-significant increase in number of reads at 37°C, suggesting they have less impact on fitness at this temperature.

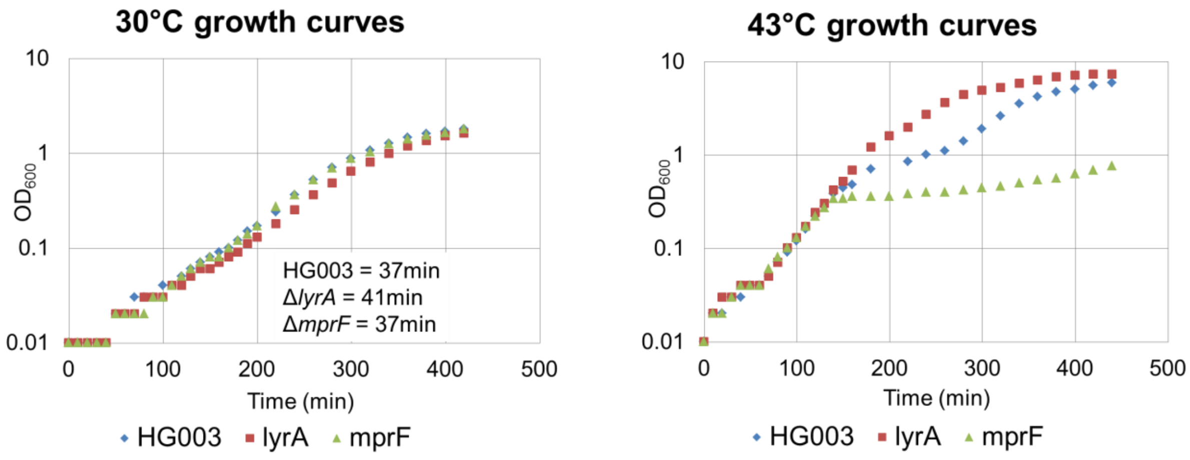

Figure S6. Validation of genes. Growth curves of Δ*lyrA* and Δ*mprF* at 30°C and 43°C. Δ*mprF* has a dramatic growth defect at high temperatures, while Δ*lyrA* grows more rapidly than wild type.

ADDITIONAL TABLES

Table S1. Gene Essentiality in HG003

Please refer to Additional File 2

Table S2. Genes important for growth at a set of temperatures

Please refer to Additional File 3

Table S3. Strains and plasmids used in this study.

**Strains**	**Genotype/Phenotype**	**Source/Reference**
RN4220	*S. aureus* subsp. aureus NCTC8325 MSSA; r− m+; partial *agr* defect	[13]
HG003	*S. aureus* subsp. aureus NCTC8325 MSSA ; Φ11 Φ12 Φ113, r+ m+; *agr+*	[14]
TM51	RN4220 attB::Orf5 (pTM378)	[1]
TM53	RN4220 attB::Orf5 (pTM381)	[1]
TM174	RN4220 (pOrf5 Tnp+) Km^R	This study
Tm175	RN4220 (pOrf5 Tnp-) Km^R	This study
TM176	HG003 (pOrf5 Tnp+) Km^R	This study
TM177	HG003 (pOrf5 Tnp-) Km^R	This study
TM222	HG003 *attLint::*FRT *attR::*FRT	This study
TM226	HG003 Φ11::FRT	This study
TM231	TM226 (pOrf5 Tnp+)	This study
TM232	TM226 (pOrf5 Tnp-)	This study
ΔlyrA	HG003 *lyrA*::Km^R	This study
ΔmprF	HG003 *mprF*::Km^R	This study
**Plasmids**		
pKFC	temperature-sensitive shuttle vector; Amp^r in *E. coli*, Cm^r in *S aureus*	[4]
pUC19	pMB1 ori *lacZ'* Ap^R	Clontech
pTM402	pT181 *repC- cop 623* ori sso; 1-kb DNA Φ 11 fragment; mini-Tnp cassette with *ermB* of Tn551 and outward facing P_{pen} promoter; Em^R	[1]
pTM378	$pWV01^{ts}$ ori *aphA-3* Gram+ RBS HMAR1 C9 transposase ; Km^R	[1]
pTM381	$pWV01^{ts}$ ori *aphA-3* Gram+ RBS ΔHMAR1 C9 truncated transposase ; Km^R	[1]
pCP20	Vector containing the *S. cervesiae* FLP recombinase	[15]
pMS182	*E. coli/S. aureus* shuttle vector Cm^r pLI50 with P_{pen} GFP-mut2	[16]
pORF5 Tnp^+	pTM378 with the cI-like repressor ORF5 from Φ11	This study
pORF5 Tnp^-	pTM381 with the cI-like repressor ORF5 from Φ11	This study
pTM239(Blunt)	pTM402 modified with NotI-P7 annealing site-ITR2::MmeI-NotI with DNA barcode (gGTAa) - Em^R	This study
pTM240(P_{pen})	pTM402 modified with NotI-P7 annealing site-ITR2::MmeI-NotI with DNA barcode (gAATa) and outward facing P_{pen} promoter- Em^R	This study
pTM241(P_{cap})	pTM402 modified with NotI-P7 annealing site-ITR2::MmeI-NotI with DNA barcode (gTGGa) for outward facing P_{cap} promoter- Em^R	This study
pTM242(P_{tuf})	pTM402 modified with NotI-P7 annealing site-ITR2::MmeI-NotI with DNA barcode (gGATa) for outward facing P_{tuf} promoter- Em^R	This study
pTM243(P_{erm})	pTM402 modified with NotI-P7 annealing site-ITR2::MmeI-NotI with DNA barcode (gGCAa) for outward facing P_{erm} promoter- Em^R	This study
pTM244(P_{Dual})	pTM402 modified with NotI-P7 annealing site-ITR2::MmeI-NotI with DNA barcode (gATTa) for outward facing P_{Dual} promoter- Em^R	This study
pTM204*attLint*	pKFC vector with 1-kb DNA homology regions flanking *attLint* on each side of FRT recombination sequence	This study
pTM204*attR*	pKFC vector with 1-kb DNA homology regions flanking *attR* on	This study

	each side of FRT recombination sequence	
pTM195	pLI50- P_{pen}-FLP recombinase of *Saccharomyces cervesiae*	This study
(pKFC-lyrA)	pKFC vector with 1-kb DNA homology regions flanking *lyrA* on each side of the kanamycin resistance cassette	[17]
(pKFC-mprF)	pKFC vector with 1-kb DNA homology regions flanking *mprF* on each side of the kanamycin resistance cassette	This study

Table S4. Oligonucleotides and primers used in this study.

Name	Sequence (5’ – 3’)
Tm1	GTCGTAAAGCTTCTTCTTGAGTACACTTTC
Tm2	GTCGTAGAATTCCACTGCTGAAATAGAC
Tm138-ITR1	AATACTCTTGAATGGCATGCCAAGTTTACTCATAACATAACAGGTTGGCTGATAAGTCCCC<br>GGTCTATAGACACATAGATTCGTGGCGCGCCATGAGTATTCATGATGAGAGTGATTGGTC<br>TTGCGTATGGTTAACCCTAAAGTTATGGAAATAAGACTTAGAAGCAAACTTAAGAGTGTGT<br>TGATAGTGCATTATCTTAAAATTTTGTATAATAGGAATTGAAGTTAAATTAGATGCTAAAAA<br>TTTGTAATTAAGAAGGAGGGATTCGACATGAACAAAAATATAAAATATTCTCAAAACTTTTT<br>AACGAGTGAAAAAGTACTCAACCAAATAAT
Tm140-ITR2 MmeI	ATATAGTTATACGCGTAATGTAGTTACTCTCAATATAGACCGGGGACTTATCATCCAACCT<br>GTTATGTTATGCGGCCGCTAGATTGATTAATTAAACAGTAGTCTGGATCCTTTGCGGGAA
Tm162	GCTTATACGCGTGCCATAACGNNNAATGTAGTTACTCTC
Tm166	CTTATACGCGTGGTCTTCGTATGCCGTCTTCTGCTTGCGGCCGCATAACTATATAGGAAC
Tm167	ATTGTACAGCGGCCGCAAGCAGAAG
Tm170	GTTATACGCAAAAGCGACTCATAG
Tm71-P1 attR	GACGGCCAGTGAATTTCCCTATTAAATGCC
Tm72-P2 attR FRT	ATTCTCTAGAAAGTATAGGAACTTCTTGCTTCAATTTGACGC
Tm73-P3 attR FRT	ATACTTTCTAGAGAATAGGAACTTCCATGAGACAATAAACG
Tm74-P4 attR	TGATTACGCCAAGCTGTAGGTTAGTTGATGAC
Tm75-P1 attL	GACGGCCAGTGAATTGATGCCAAACTAGCTG
Tm76-P2 attL FRT	ATTCTCTAGAAAGTATAGGAACTTCTTACGTTTGAATTGTTTC
Tm77-P3 attL FRT	ATACTTTCTAGAGAATAGGAACTTCGTTAAAGAACATGGCC
Tm78-P4 attL	TGATTACGCCAAGCTCCATATTCGGATAGTAC
mprF1	TTCATGGATCCGCACTTCATTTCACCAAATATCATGG
mprF2	ATCACCTCAAATGGTTCGCCCTGATTCATTTTTTCACATC
mprF3	GATGTGAAAAAATGAATCAGGGCGAACCATTTGAGGTGAT
mprF4	GCATAACTGTATACCTCCTAGGTACTAAAACAATTCAT
mprF5	ATGAATTGTTTTAGTACCTAGGAGGTATACAGTTATGC
mprF6	AAAACAGTCGACCCTACCTTATATCTTC
lyrA1	GATCTGATTGGATCCCATAATGACATCATGTTCATGTAC
lyrA2	CCTATCACCTCAAATGGTTCGCTATATGTAACCTCCATTAGG
lyrA3	CCTAATGGAGGTTACATATAGCGAACCATTTGAGGTGATAGG
lyrA4	GAGGTACTAGCAAGCGCTTTGTCCTAGGTACTAAAACAATTCATC
lyrA5	GATGAATTGTTTTAGTACCTAGGACAAAGCGCTTGCTAGTACCTC
lyrA6	GATCTGATTGTCGACGCAGAGCGCATCGGTCTTTTCG
Tm79	CTTATCGGATCCAAGAAGGAGATATACATATGCCACAATTTGG
Tm80	CTTAGAGGCGCGCCTTATATGCGTCTATTTATG
Tm179	GCCGTCTTCTGCTTGGAATTCACTGGCCGTCG
Tm180	GGTCGCCGTATCATTAAGCTTGGCGTAATCATG
TM196	CAAGCAGAAGACGGCATACGAAGACC
TM198	CGCTCACCCAAATATATATCTTGATG
TM58	CAAGGTCATAAAGCTTCATTTG
TM59	GATTGAATCGCCTTATGCATG
TM214	5Biotin-TEG/CGTTAGTAACCTTGCGATGTC
TM215	GGCCCAACGTGAATCGACATCGCAAGGT
LIB_AdaptT_1_long	TTCCCTACACGACGCTCTTCCGATCT**AGTCATGC**NN
LIB_AdaptT_2_long	TTCCCTACACGACGCTCTTCCGATCT**ACGTACTG**NN
LIB_AdaptT_3_long	TTCCCTACACGACGCTCTTCCGATCT**TGACTGCA**NN
LIB_AdaptT_4_long	TTCCCTACACGACGCTCTTCCGATCT**TCGACGAT**NN
LIB_AdaptT_5_long	TTCCCTACACGACGCTCTTCCGATCT**CTAGCATG**NN
LIB_AdaptT_6_long	TTCCCTACACGACGCTCTTCCGATCT**GACTGTAC**NN
LIB_AdaptB_1_long	**GCATGACT**AGATCGGAAGAGCGTCGTGTAGGGAA
LIB_AdaptB_2_long	**CAGTACGT**AGATCGGAAGAGCGTCGTGTAGGGAA
LIB_AdaptB_3_long	**TGCAGTCA**AGATCGGAAGAGCGTCGTGTAGGGAA

LIB_AdaptB_4_long	**ATCGTCGA**GATCGGAAGAGCGTCGTGTAGGGAA
LIB_AdaptB_5_long	**CATGCTAG**AGATCGGAAGAGCGTCGTGTAGGGAA
LIB_AdaptB_6_long	**GTACAGTC**AGATCGGAAGAGCGTCGTGTAGGGAA
Tm199 –Final PCR 5	CAAGCAGAAGACGGCATACGAAGACCACGCGTGCCATAAC
LIB_PCR_3 – Final PCR 3	AATGATACGGCGACCACCGAACACTCTTTCCCTACACGACGCTCTTCCGATCT

1. Wang H, Claveau D, Vaillancourt JP, Roemer T, Meredith TC: **High-frequency transposition for determining antibacterial mode of action.** *Nat Chem Biol* 2011, **7:**720-729.
2. Kilby NJ, Snaith MR, Murray JA: **Site-specific recombinases: tools for genome engineering.** *Trends Genet* 1993, **9:**413-421.
3. Storici F, Coglievina M, Bruschi CV: **A 2-microm DNA-based marker recycling system for multiple gene disruption in the yeast Saccharomyces cerevisiae.** *Yeast* 1999, **15:**271-283.
4. Kato F, Sugai M: **A simple method of markerless gene deletion in Staphylococcus aureus.** *J Microbiol Methods* 2011, **87:**76-81.
5. Pitcher DG, Saunders NA, Owen RJ: **Rapid Extraction of Bacterial Genomic DNA with Guanidium Thiocyanate.** *Letters in Applied Microbiology* 1989, **8:**151-156.
6. Lis JT, Schleif R: **Size Fractionation of Double-Stranded DNA by Precipitation with Polyethylene-Glycol.** *Nucleic Acids Res* 1975, **2:**383-389.
7. Georgiou CD, Papapostolou I, Grintzalis K: **Protocol for the quantitative assessment of DNA concentration and damage (fragmentation and nicks).** *Nat Protoc* 2009, **4:**125-131.
8. Ralser M, Querfurth R, Warnatz HJ, Lehrach H, Yaspo ML, Krobitsch S: **An efficient and economic enhancer mix for PCR.** *Biochemical and Biophysical Research Communications* 2006, **347:**747-751.
9. Goodman AL, Wu M, Gordon JI: **Identifying microbial fitness determinants by insertion sequencing using genome-wide transposon mutant libraries.** *Nat Protoc* 2011, **6:**1969-1980.
10. Hamady M, Walker JJ, Harris JK, Gold NJ, Knight R: **Error-correcting barcoded primers for pyrosequencing hundreds of samples in multiplex.** *Nat Methods* 2008, **5:**235-237.
11. Buschmann T, Bystrykh LV: **Levenshtein error-correcting barcodes for multiplexed DNA sequencing.** *BMC Bioinformatics* 2013, **14:**272.
12. Pritchard JR, Chao MC, Abel S, Davis BM, Baranowski C, Zhang YJ, Rubin EJ, Waldor MK: **ARTIST: high-resolution genome-wide assessment of fitness using transposon-insertion sequencing.** *PLoS Genet* 2014, **10:**e1004782.
13. Kreiswirth BN, Lofdahl S, Betley MJ, O'Reilly M, Schlievert PM, Bergdoll MS, Novick RP: **The toxic shock syndrome exotoxin structural gene is not detectably transmitted by a prophage.** *Nature* 1983, **305:**709-712.
14. Herbert S, Ziebandt AK, Ohlsen K, Schafer T, Hecker M, Albrecht D, Novick R, Gotz F: **Repair of global regulators in Staphylococcus aureus 8325 and comparative analysis with other clinical isolates.** *Infect Immun* 2010, **78:**2877-2889.
15. Cherepanov PP, Wackernagel W: **Gene disruption in Escherichia coli: TcR and KmR cassettes with the option of Flp-catalyzed excision of the antibiotic-resistance determinant.** *Gene* 1995, **158:**9-14.
16. Swoboda JG, Meredith TC, Campbell J, Brown S, Suzuki T, Bollenbach T, Malhowski AJ, Kishony R, Gilmore MS, Walker S: **Discovery of a small molecule that blocks wall teichoic acid biosynthesis in Staphylococcus aureus.** *ACS Chem Biol* 2009, **4:**875-883.

17. Santa Maria JP, Jr., Sadaka A, Moussa SH, Brown S, Zhang YJ, Rubin EJ, Gilmore MS, Walker S: **Compound-gene interaction mapping reveals distinct roles for Staphylococcus aureus teichoic acids.** *Proc Natl Acad Sci U S A* 2014, **111:**12510-12515.
